# AtxA-Controlled Small RNAs of *Bacillus anthracis* Virulence Plasmid pXO1 Regulate Gene Expression *in trans*

**DOI:** 10.3389/fmicb.2020.610036

**Published:** 2021-01-15

**Authors:** Ileana D. Corsi, Soumita Dutta, Ambro van Hoof, Theresa M. Koehler

**Affiliations:** ^1^Department of Microbiology and Molecular Genetics, McGovern Medical School, The University of Texas Health Science Center at Houston, Houston, TX, United States; ^2^MD Anderson Cancer Center UTHealth Graduate School of Biomedical Sciences, The University of Texas, Houston, TX, United States

**Keywords:** anthrax, transcription, sRNA, RNA-seq, *Bacillus*, gene expression, plasmid, *anthracis*

## Abstract

Small regulatory RNAs (sRNAs) are short transcripts that base-pair to mRNA targets or interact with regulatory proteins. sRNA function has been studied extensively in Gram-negative bacteria; comparatively less is known about sRNAs in Firmicutes. Here we investigate two sRNAs encoded by virulence plasmid pXO1 of *Bacillus anthracis*, the causative agent of anthrax. The sRNAs, named “XrrA and XrrB” (for pXO1-encoded regulatory RNA) are abundant and highly stable primary transcripts, whose expression is dependent upon AtxA, the master virulence regulator of *B. anthracis*. sRNA levels are highest during culture conditions that promote AtxA expression and activity, and sRNA levels are unaltered in Hfq RNA chaperone null-mutants. Comparison of the transcriptome of a virulent Ames-derived strain to the transcriptome of isogenic sRNA-null mutants revealed multiple 4.0- to >100-fold differences in gene expression. Most regulatory effects were associated with XrrA, although regulation of some transcripts suggests functional overlap between the XrrA and XrrB. Many sRNA-regulated targets were chromosome genes associated with branched-chain amino acid metabolism, proteolysis, and transmembrane transport. Finally, in a mouse model for systemic anthrax, the lungs and livers of animals infected with *xrrA*-null mutants had a small reduction in bacterial burden, suggesting a role for XrrA in *B. anthracis* pathogenesis.

## Introduction

Eukaryotic and bacterial organisms have evolved mechanisms employing specialized RNA molecules to modulate gene expression. The microRNAs (miRNAs) of eukaryotic cells are non-coding RNAs of 21 to 24 nucleotides that serve as mRNA-targeting templates to guide the function of miRNA-associated proteins (Grishok et al., 2001; Song et al., 2003; Vaucheret et al., 2004). Upon target recognition, these proteins, collectively referred to as Argonaute proteins, induce mRNA decay or inhibit mRNA translation initiation (Pillai et al., 2004; Filipowicz et al., 2005; Wu and Xie, 2006). Similar to miRNAs, bacterial cells employ small regulatory RNAs (sRNAs) between 50 and 500 nucleotides long to modulate gene expression by direct base-pairing with target mRNAs or direct interaction with regulatory proteins (Gottesman and Storz, 2011). sRNAs base-pair with mRNAs using short complementary sequences, leading to modulation of transcription termination, translation initiation, and/or mRNA decay. Protein-interacting sRNAs mimic specific nucleic acid structures or sequence motifs recognized by RNA- or DNA-binding regulators, resulting in titration of such regulators from their normal target sites.

Similar to miRNA association with Argonaute proteins in eukaryotes to affect function, mRNA-targeting sRNAs of bacteria can partner with RNA-binding proteins. The most-well studied RNA chaperone that participates in sRNA-mediated regulation is the hexameric Hfq protein (Wassarman et al., 2001; Møller et al., 2002). Hfq forms a donut-like structure with three RNA-binding surfaces, binding an sRNA and its cognate mRNA target at separate faces of the protein and allowing complementary sequences to come into proximity and base-pair at the rim of the protein (Schumacher et al., 2002; Link et al., 2009; Panja et al., 2013). In many bacterial species, deletion of *hfq* results in shortened half-lives of sRNAs and impaired sRNA-mediated regulation (Vytvytska et al., 1998; Sledjeski et al., 2001; Lenz et al., 2004; Deng et al., 2012; Santiago-Frangos et al., 2016).

Mounting evidence suggests that sRNAs can function as important virulence regulators in a number of bacterial pathogens. In *Pseudomonas aeruginosa*, a Gram-negative pathogen, two RNA-binding proteins, RsmA and RsmF, positively regulate genes involved in planktonic lifestyle while repressing genes involved in biofilm formation. Several sRNAs of the Rsm system titrate RsmA and RsmF to allow induction of biofilm formation (Vakulskas et al., 2015; Miller et al., 2016; Janssen et al., 2018). sRNAs also control virulence in some Gram-positive bacteria. An Hfq-independent sRNA of *Staphylococcus aureus*, RNAIII, allows translation of the mRNA encoding alpha-hemolysin while inhibiting expression of the repressor of toxin (*rot*) gene. In *Listeria monocytogenes*, the LhrA sRNA base-pairs to and represses translation of the *chiA* mRNA, which encodes chitinase A (Nielsen et al., 2011). Mutants deleted for *chiA* are defective in liver and spleen colonization of mice in a listeriosis model (Chaudhuri et al., 2013). Hfq stabilizes LhrA and facilitates the base-pairing interaction with *chiA*.

Knowledge of sRNA-mediated regulation is limited in the *Bacillus cereus sensu lato* species, a group of closely-related Gram-positive pathogens. Only one sRNA has been described in detail. In the YBT-1518 strain of the insect and nematode pathogen *Bacillus thuringiensis*, the BtsR1 sRNA silences expression of a cry toxin, which mediates killing of the host, by inhibiting translation initiation (Peng et al., 2018). Bioinformatic analysis of microarray data obtained from *Bacillus cereus* strain ATCC14579 grown in AgNO_3_ stress predicts expression of several hundred sRNAs from intergenic regions along the genome (Sridhar and Gayathri, 2019). However, whether these sRNAs are functional remains unknown. *B. thuringiensis* and the mammalian pathogen *B. anthracis* harbor homologs of the SR1 sRNA found in multiple *Bacillus* species (Gimpel et al., 2012). *In vitro* transcribed *B. thuringiensis* SR1 was found to interact with a homolog of the *Bacillus subtilis ahrC* transcript, which encodes a regulator that activates transcription of arginine catabolism operons (Heidrich et al., 2006; Gimpel et al., 2012). Interaction between *B. anthracis* SR1 and the *ahrC* homolog of this bacterium has not been experimentally confirmed.

Here, we report the first investigation of sRNA function in *B. anthracis*, the causative agent of anthrax. The *B. anthracis* genome is comprised of a 5-Mbp circular chromosome and two virulence plasmids, pXO1 and pXO2, 182 and 94 kbp in length, respectively. pXO1 carries the three anthrax toxin genes *pagA* (protective antigen), *cya* (edema factor), and *lef* (lethal factor), as well as *atxA*, encoding the *trans*-acting anthrax toxin activator AtxA. pXO2 carries the glutamic-acid-capsule biosynthesis operon *capBCADE* and genes for two functional paralogs of AtxA, the proteins AcpA and AcpB. AtxA positively regulates expression of the three toxin genes as well as the *acpA* gene (Dai et al., 1995; Drysdale et al., 2004). In turn, the AcpA protein positively regulates expression of the capsule biosynthesis operon, which aids in immune evasion (Drysdale et al., 2005). Read-through of a weak terminator hairpin at the end of the capsule biosynthesis operon transcript leads to transcription of the downstream *acpB* gene, which in turn positively regulates the operon in a feed-forward loop (Drysdale et al., 2005). The AtxA protein and its paralogs are members of an emerging class of virulence regulators designated PRD-containing virulence regulators (PCVRs), so termed due to the presence of phosphoenolpyruvate phosphotransferase system - regulated domains (PRDs) in their protein structures (Hondorp et al., 2013; Raynor et al., 2018).

A study comparing the transcriptome of the attenuated *B. anthracis* strain Ames 35 (pXO1^+^, pXO2^–^) to an isogenic *atxA*-null mutant revealed two unannotated transcripts encoded by pXO1. The transcripts, initially designated sRNA1 and sRNA2, were highly expressed in the parent strain and showed greatly reduced expression in the *atxA* mutant (McKenzie et al., 2014). Our own recent RNA-seq analysis confirmed expression of these transcripts in the fully virulent *B. anthracis* Ames strain (pXO1^+^, pXO2^+^) (Raynor et al., 2018). Culture *of B. anthracis* in toxin-inducing conditions resulted in high level expression of the transcripts. Expression of both putative sRNAs was abolished in an Δ*atxA*Δ*acpA*Δ*acpB* strain and complementation of exogenously expressed AtxA restored sRNA expression (Raynor et al., 2018). The pXO1 loci of the sRNAs and the tight regulation by AtxA suggest a relationship between sRNA-mediated regulation and virulence.

Of further interest, while about half of all bacterial genomes sequenced contain an *hfq* gene (Sun et al., 2002), and most of those species harbor a single copy of *hfq* in the chromosome, *B. anthracis* has three Hfq homologs. Two of the *hfq* genes, *hfq1* and *hfq2*, are located on the chromosome. The third *hfq* gene, *hfq3*, is located on pXO1, the plasmid encoding the predicted sRNAs. Hfq2 and Hfq3, but not Hfq1, are functional when expressed in *Escherichia coli* (Vrentas et al., 2015). Hfq2 and Hfq3 form hexamers *in vitro*, while Hfq1 purifies as a monomer (Vrentas et al., 2015). Our *B. anthracis* RNA-seq data indicate that the chromosome-borne *hfq2* is positively regulated by AtxA (Raynor et al., 2018). AtxA-mediated positive control of both the sRNAs and Hfq2, and the predicted functionality of Hfq2 and Hfq3, suggest roles for the sRNAs and Hfq proteins in *B. anthracis* gene regulation.

In this study, we designated the sRNAs previously described as sRNA2 and sRNA1 (McKenzie et al., 2014) as “XrrA” and “XrrB” (for pXO1-encoded regulatory RNA). We mapped the loci and assessed expression and function of XrrA and XrrB in *B. anthracis.* We constructed deletion mutants Δ*xrrA*, Δ*xrrB*, and Δ*xrrA*Δ*xrrB* in an Ames strain background and compared gene expression of these mutants to that of the Ames-derived parent strain using RNA-seq. We tested for effects of the three Hfq chaperones of *B. anthracis* on sRNA half-life and assessed virulence of sRNA mutant strains using a murine model of systemic anthrax. XrrA and XrrB are the first regulatory RNAs experimentally examined in *B. anthracis* and are shown here to regulate metabolic and virulence gene expression in this pathogen.

## Materials and Methods

### Growth Conditions

*Escherichia coli* strains were grown in 5 ml of Luria-Bertani (LB) (Bertani, 1951) broth at 30 or 37°C, as indicated. For RNA isolation, *B. anthracis* strains were cultured in 25 ml of brain heart infusion (BHI, Becton, Dickson and Company, Franklin Lakes, NJ, United States) broth at 30°C overnight before sub-culturing into 25 ml of casamino acids medium supplemented with 0.1% w/v of glucose (CA) (Thorne and Belton, 1957; Hadjifrangiskou et al., 2007) or LB medium at a starting OD_600_ of 0.08. *B. anthracis* sub-cultures were grown at 37°C in air (CA-Air or LB-Air) or in 5% atmospheric CO_2_ (CA-CO_2_ or LB-CO_2_; medium supplemented with 0.8% sodium bicarbonate). All cultures were incubated with shaking at 200 r.p.m. Expression of recombinant XrrA in the Δ*xrrA* strain was induced by addition of 500 μM IPTG to the medium at early exponential phase. For growth curves, *B. anthracis* strains were sub-cultured at a starting OD_600_ of 0.08 into a 26-well plate containing 1 ml of CA medium per well. Cultures were grown in a Biotek SynergyH1 microplate reader (Biotek, Winooski, VT, United States) at 37°C in 5% atmospheric CO_2_ with continuous orbital shaking at 355 c.p.m. Absorbance measurements were taken hourly for 18 h. LB agar was used for growth of all strains on solid media. Media contained antibiotics when appropriate: carbenicillin (100 μg ml^–1^) and erythromycin (150 μg ml^–1^) for *E. coli*, and erythromycin (10 μg ml^–1^), and spectinomycin (100 μg ml^–1^) for *B. anthracis*.

### Strain Construction

Strains used in this study are listed on Table 1. Oligonucleotide primers used in polymerase chain reactions (PCR) are listed in Supplementary Table 1. The fully virulent Ames strain (pXO1^+^, pXO2^+^), the Ames-derived UTA37, and isogenic mutants of these strains were used to determine the effect of *atxA* and *acpA* deletions on XrrA and XrrB expression, the effect of deleting *xrrA* and *xrrB* on the transcriptome of *B. anthracis*, and for validation of XrrA-mediated control of the *inhA1* target. The Ames-derived parent strain UTA37 contains a recombinant *atxA* gene at the native pXO1 locus that encodes a functional AtxA protein with a FLAG tag at the C-terminus of the protein. This strain has been used to monitor AtxA protein levels, and AtxA expression and function is unchanged from Ames (Raynor et al., 2018). The attenuated ANR-1 strain (pXO1^+^, pXO2^–^) and isogenic mutants were used for all other experiments. *E. coli* strains TG1 and GM2163 were hosts for general cloning and plasmid constructions using standard methods (Marrero and Welkos, 1995).

**TABLE 1:** *Bacillus anthracis* strains and plasmids.

Strains or Plasmids	Relevant characteristics^a,b^	Source
**Strains**		
Ames	*B. anthracis* (pXO1^+^, pXO2^+^)	Ivins et al., 1990
UTA5	Ames-derivative, *inhA1::specR*	Pflughoeft et al., 2014
UTA22	Ames-derivative, ΔatxA	Dale et al., 2018
UTA37	Ames-derivative with *atxA-FLAG* expressed from the native locus	Raynor et al., 2018
UTA38	UTA37-derivative, Δ*xrrB*	This work
UTA39	UTA37-derivative, Δ*xrrA*	This work
UTA41	UTA37-derivative, Δ*xrrA*Δ*xrrB*	This work
UTA44	Ames-derivative, Δ*acpA*	Raynor et al., 2018
ANR-1	*B. anthracis* (pXO1^+^, pXO2^–^)	Welkos et al., 2001
UT434	ANR-1-derivative, Δ*xrrB*	This work
UT435	ANR-1-derivative, Δ*xrrA*	This work
UT436	ANR-1-derivative, Δ*xrrA*Δ*xrrB*	This work
UT440	ANR-1-derivative, Δ*hfq1*	This work
UT437	ANR-1-derivative, Δ*hfq2*	This work
UT438	ANR-1-derivative, Δ*hfq3*	This work
UT471	ANR-1-derivative, Δ*hfq1*Δ*hfq2*Δ*hfq3*	This work
**Plasmids**		
pHY304	Heat-sensitive vector used for deletion of indicated loci by homologous recombination; Erm^r^	Ho et al., 1989
pUTE657	Expression vector with IPTG-inducible *lac* operon promoter; Spec^r^	Pflughoeft et al., 2011
pUTE1205	pUTE657-derived expression vector containing *xrrA* sequence under the control of the *lac* operon promoter; Spec^r^	This work

*^*a*^Δ mutants were created by markerless deletion of coding sequences using pHY304-derived constructs.*

*^*b*^Erm^*r*^ indicates resistance to erythromycin*.

Markerless sRNA deletions (Δ*xrrA*, Δ*xrrB*, Δ*xrrA*Δ*xrrB*) in the Ames and ANR-1 backgrounds, and *hfq* deletions (Δ*hfq1*, Δ*hfq2*, Δ*hfq3*, Δ*hfq1*Δ*hfq2*Δ*hfq3*) in the ANR-1 background were made by homologous recombination as described previously (Ho et al., 1989; Raynor et al., 2018). Briefly, the pHY304 plasmid contains a heat-sensitive origin of replication and an erythromycin resistance marker to select for allelic recombination (Chaffin et al., 2005). Two-kb DNA constructs containing target gene flanking sequences 1-kb upstream and 1-kb downstream from the gene locus were created using PCR and gene-specific primers listed in Supplementary Table 1. The resulting plasmids were electroporated into *B. anthracis*. Plasmid-containing isolates were grown in 25 ml of BHI broth at the non-permissive temperature of 41°C in the presence of erythromycin. Cultures were sub-cultured into 25 ml of fresh BHI broth with no erythromycin at a starting OD_600_ of 0.08 and grown at 30°C. Deletion mutants were verified by PCR and sequencing. The Δ*xrrA*Δ*xrrB* and Δ*hfq1*Δ*hfq2*Δ*hfq3* mutants were created by sequentially deleting each gene using one pHY304-derived shuttle vector at a time. Gene deletions were confirmed using PCR.

To create a Δ*xrrA*-null mutant complemented with *xrrA*, the gene was amplified from the native locus using PCR with *xrrA*-specific primers, and ligated into pUTE657 downstream of the *lac* operon promoter such that *xrrA* transcription is IPTG-inducible. The resulting construct, named pUTE1205, was electroporated into the Δ*xrrA* Ames strain, creating strain UTA39 (pUT1205). Plasmid-containing isolates were confirmed by PCR using pUTE657-specific primers.

Newly-created mutants were grown in 25 ml of Phage Assay (PA) broth at 30°C for 72 h to induce spore formation, and spores were prepared as previously described (Thorne, 1968). Spores were finally stored at 4°C in a suspension of 5 ml of sterile water and strain names were assigned as listed on Table 1.

### RNA Isolation

RNA isolation was performed as described previously (Raynor et al., 2018). Briefly, *B. anthracis* strains were cultured in CA-CO_2_, CA-Air, LB-CO_2_, or LB-Air as indicated. At early stationary phase (OD_600_ = 1.0–1.5), cells from 10-ml samples were centrifuged at 10,000 × *g* for 15 min. Cell pellets were resuspended in 500 μl of PBS followed by an equivalent volume of saturated acid phenol (pH 4.3) (Fisher Bioreagents, Fair Lawn, NJ, United States) at 65°C. Samples were homogenized in screw cap tubes containing 400 μl of 0.1-mm diameter zirconia/silica beads (BioSpec Products, Bartlesville, OK, United States) by bead beating in a Mini Beadbeater (BioSpec Products, Bartlesville, OK, United States). Samples were beaten twice for 1 min, with a 5-min incubation at 65°C between homogenizations. Samples were centrifuged at 16,000 × *g* for three min at 4°C. The aqueous phase was collected and the phenol extraction repeated. Following centrifugation, the aqueous phase was mixed with one-third volume of chloroform and incubated at room temperature for 10 min prior to centrifugation at 16,000 × *g* for 15 min at 4°C. To precipitate the RNA from the final aqueous phase, 20 ng of glycogen, one-tenth volume of 3 M sodium acetate, and 3 volumes of 100% ice-cold ethanol were added to the samples. RNA was precipitated at −80°C for one h to overnight. Samples were centrifuged at 16,000 × *g* for 30 min at 4°C. The resulting pellet was washed twice with ice-cold 75% ethanol before resuspending in DEPC-treated sterile water. RNA was quantified using a Nanodrop Spectrophotometer ND-1000.

### Northern Blotting

Expression of XrrA, XrrB, 5S rRNA load control, 16S rRNA, 23S rRNA, and *rpsO* mRNA was determined using northern blot analysis. To detect XrrA, XrrB, 5S rRNA, and *rpsO* mRNA 3–10 μg of total RNA was denatured for 5 min at 95°C in Gel Loading Buffer II (Invitrogen, Carlsbad, CA, United States) and subjected to electrophoresis on an 8% polyacrylamide – 8 M urea gel. To determine the length of the sRNAs, 12 ng of biotinylated sRNA ladder (Kerafast, Boston, MA, United States) was added to the first lane of the gel. To detect 16S rRNA and 23S rRNA, 3 μg of total RNA was denatured as described above and subjected to electrophoresis on a 1% agarose – 1.5% formaldehyde gel. Size-separated RNA was then transferred to an Amersham Hybond-N nylon membrane (GE Healthcare, Little Chalfront, United Kingdom) via capillary transfer in 50 mM NaOH. After overnight transfer, RNA was crosslinked to the membrane by UV light exposure and subsequently incubated in NorthernMax Prehybridization/Hybridization Buffer (Invitrogen, Carlsbad, CA, United States) at 42°C. Biotinylated DNA probes were used to detect expression of the RNAs. The probes were generated by PCR using the indicated primers (Supplementary Table 1) and the resulting double-stranded DNA templates were subjected to random biotin-labeling by incorporation of biotin-11-dUTP with Klenow enzyme (Thermo Scientific, Waltham, MA, United States). Hybridization of RNA with the respective biotin-labeled DNA probes was detected using the North2South Chemiluminescent Hybridization and Detection Kit (Thermo Scientific, Waltham, MA, United States) according to manufacturer’s instructions.

### 5′ and 3′ Rapid Amplification of cDNA Ends (RACE)

RACE analysis was performed as described previously with modifications (Bensing et al., 1996; Argaman et al., 2001). RNA Adapter sequences and oligonucleotide primers used in RACE are listed in Supplementary Table 1. To precisely map the 5′ and 3′ ends of XrrA and XrrB, RNA samples isolated from ANR-1 were treated with 30 units of DNase I (New England Biolabs, Ipswich, MA, United States) for 30 min at 37°C to remove potential DNA contamination. RNA was recovered from the DNAase treatment and all subsequent reactions using the RNA Clean and Concentrator Kit (Zymo Research, Irvine, CA, United States), according to kit instructions.

For 5′ RACE, 5-μg samples of RNA were treated with 20 units of RNA 5′ Polyphosphatase (5′PP, Lucigen, Middleton, WI, United States) at 37°C for 1 h. This reaction removes the 5′ triphosphate of primary RNA transcripts, leaving a 5′ monophosphate which is then ligated to the 3′-OH group of the 5′ RNA Adapter. The treated RNA was ligated to the 5′ RNA Adapter by incubation with 500 pmoles of the adapter, 100 units of T4 RNA Ligase I (New England Biolabs, Ipswich, MA, United States) and 150 μM ATP at 16°C overnight. One microgram of the resulting ligated RNA was used to synthesize cDNA using a locus-specific internal primer for XrrA or XrrB and the Super Script III Reverse Transcriptase Kit (Invitrogen, Carlsbad, CA, United States). Finally, an aliquot of the cDNA synthesis reaction was used to perform PCR using the same locus-specific primer for either XrrA or XrrB and a 5′ Adapter-specific primer. PCR products were visualized by 1.5% agarose gel electrophoresis. Products of interest were excised from the gel and recovered using the DNA Clean and Concentrator Kit (Zymo Research, Irvine, CA, United States), according to kit instructions. Recovered PCR products were assessed using Sanger sequencing employing both PCR primers (Genewiz, South Plainfield, NJ, United States).

For 3′ RACE, 15-μg samples of RNA were treated with 20 units of Calf Intestinal Alkaline Phosphatase (CIP, New England Biolabs, Ipswich, MA, United States) for one h at 37°C. This reaction removes 3′ phosphate groups from the ends of RNA. The 3′-OH group of RNA in the sample was ligated to the 3′ RNA Adapter, which contains a 5′ monophosphate modification. The adapter also contains an inverted deoxythymidine at the 3′ to prevent self-ligation of the adapter during the ligation reaction. CIP-treated RNA was incubated with 500 pmol of the 3′ RNA Adapter, 100 units of T4 RNA ligase and 150 μM ATP at 16°C overnight. One microgram of the resulting RNA was used to perform cDNA synthesis, using an adapter-specific primer for first strand synthesis with the Super Script III Reverse Transcriptase Kit (Invitrogen, Carlsbad, CA, United States). Finally, an aliquot of the cDNA synthesis reaction was used to perform a PCR, using the 3′ RNA Adapter-specific primer and a gene-specific internal primer for either XrrA or XrrB. PCR products were visualized and sequenced as described above.

### Determination of XrrA and XrrB 5′ End Phosphate Groups

Primary transcripts are 5′ tri-phosphorylated, while processed secondary transcripts are 5′ mono-phosphorylated. To determine if XrrA and XrrB are primary transcripts, total RNA isolated from ANR-1 was subjected to RNA 5′ Polyphosphatase treatment as described above followed by Terminator Exonuclease (TEX, Lucigen, Middleton, WI, United States) treatment, in which 3-μg samples of RNA were incubated with one unit of TEX enzyme at 30°C for 3 h. TEX specifically degrades RNA with a 5′ monophosphate. Total RNA from ANR-1 was divided into four treatment groups. The control sample TEX^–^ 5′PP^–^ received no treatments. The TEX^+^ 5′PP^–^ sample was treated with TEX alone, while the TEX^–^ 5′PP^+^ sample was treated with RNA 5′ Polyphosphatase alone. Finally, the TEX^+^ 5′PP^+^ sample was first treated with RNA 5′ Polyphosphatase, followed by TEX treatment. All RNA was recovered from reactions using the RNA Clean and Concentrator Kit (Zymo Research, Irvine, CA, United States), according to the manufacturer’s instructions. RNA samples were subjected to northern blot analysis as described above. sRNA signal was normalized to the TEX-resistant 5S rRNA signal (Patrick et al., 2009; Ferrara et al., 2017). Relative sRNA levels were calculated as a fraction of the TEX^–^ 5′PP^–^ control sample. The experiment was performed in triplicate and an analysis of variance (ANOVA) paired with Tukey’s multiple comparison analysis was used to determine significance between treatments, with an adjusted *p*-value cut-off of 0.05. As a control for TEX-mediated degradation, levels of the TEX-sensitive 16S rRNA and 23S rRNA (Fleischmann and Rocha, 2018; Lalaouna et al., 2019) in the treated samples were detected by northern blotting as described above.

### Construction of Next-Generation Sequencing (NGS) Libraries and RNA Sequencing

Total RNA from Ames strains UTA37 (Parent), UTA38 (Δ*xrrB*), UTA39 (Δ*xrrA*), UTA41 (Δ*xrrA*Δ*xrrB*) was isolated from cultures grown in CA-CO_2_ in triplicate, using saturated phenol: chloroform extraction as described above. RNA was quantified using a QubitFluorometer (Thermo Scientific, Waltham, MA, United States) and RNA quality was checked using an Agilent 2100 Bioanalyzer (Agilent Technologies, Santa Clara, CA, United States). One microgram of pure and quality-checked RNA from each sample was subjected to rRNA removal using the Ribo-Zero kit (Epicentre, Madison, WI, United States), according to manufacturer’s instructions. The rRNA-free RNA was fragmented into ∼200–400 nt fragments using divalent cations and heat (Breslow and Huang, 1991). The fragments were primed with random hexamers and Superscript II reverse transcriptase enzyme (Invitrogen, Carlsbad, CA, United States) was used for first strand synthesis of cDNA. Double-stranded cDNA was generated using DNA polymerase I and remaining RNA was removed by RNase treatment. The 5′ ends of the ds-cDNA were phosphorylated using T4 polynucleotide kinase (New England Biolabs, Ipswich, MA, United States) and the 3′ ends were adenylated using Taq enzyme (New England Biolabs, Ipswich, MA, United States). The cDNA end modifications allow for ligation to double stranded Tru-seq Illumina Adapters, which contain monophosphate modifications at the 5′ ends and thymidine overhangs at the 3′ ends. The Adapters contain short sequences for binding to the sequencer flow cell oligos and indexing of pooled libraries. They also contain sequences for primers used for PCR enrichment of the library and to initiate sequencing by synthesis. The quality of the prepared libraries was verified using an Agilent 2100 Bioanalyzer before loading the libraries into the flow cell of a NextSeq550 sequencer. Sequencing by synthesis was performed to generate 75 bp paired-end reads. Two 130M-read sequencing runs were performed and an average of 31M reads per sample was obtained.

### RNA Sequencing Bioinformatic Analysis

All bioinformatic analysis was performed using the publicly available Galaxy web resource^1^ (Afgan et al., 2018). Triplicate paired-end fastq.gz raw read files for each strain for each of the two sequencing runs were uploaded to Galaxy and converted into fastqsanger files using FASTQ Groomer (Blankenberg et al., 2010). This conversion allows read files to be processed and analyzed on Galaxy. The quality of the reads was assessed using FastQC (Andrews, 2010). Low quality bases and Illumina Adapter sequences were removed using Trim Galore! Galaxy Version 0.6.3 (Krueger, 2012). A second round of FastQC analysis was done to verify the removal of low-quality bases and Illumina Adapter sequences. Trimmed reads were aligned to the complete Ames Ancestor genome (NCBI accession numbers AE017334.2 for chromosome, AE017336.2 for pXO1, and AE017335.3 for pXO2) using the GCF_000008445.1_ASM844v1_genomic.fna FASTA file obtained from the NCBI website. Bowtie2 Galaxy Version 2.3.4.3 (Langmead and Salzberg, 2012) with default parameters was used to align the paired-end reads to the genome. On average, 97% of all the reads in each sample mapped to the reference genome. BAM files obtained from mapped reads from each of the two sequencing runs were pooled using Convert, Merge, Randomize Galaxy Version 2.4.0.0 (Barnett et al., 2011), resulting in a single BAM file per triplicate per strain. The Cufflinks/Cuffcompare/CuffDiff pipeline (Trapnell et al., 2010) was used for transcript assembly using the GCF_000008445.1_ASM844v1_genomic.gff reference annotation file from NCBI, count the number of reads mapped to each annotated transcript, and calculate differential transcript expression between the strains. The Fragments Per Kilobase of transcript per Million mapped reads (FPKM) values per assembled transcript were obtained and differential expression was calculated as the log2 (fold-change) in FPKM values between the parent strain and each mutant strain, with an adjusted p-value cut-off for significance of 0.01. The Cufflinks/Cuffcompare/CuffDiff pipeline had a lower limit of 0.00005 for *p*-value reporting. Genes differentially regulated in the Δ*xrrA* and Δ*xrrA*Δ*xrrB* mutants with a fold-change of ≥4.0 compared to the parent strain were assigned gene ontology terms based on annotation of the encoded proteins according to the UniProt web database (The UniProt Consortium, 2019). BamCoverage Galaxy Version 3.3.2.0.0 (Ramírez et al., 2016) was used to create BigWig files for visualization of read coverage over the genome on Integrative Genomics Viewer (IGV) (Thorvaldsdóttir et al., 2013). The RNA-seq data discussed in this publication have been deposited in the NCBI Gene Expression Omnibus (GEO) (Edgar et al., 2002) and are accessible through GEO Series accession number GSE152356^2^.

### Validation of XrrA-Mediated inhA1 Regulation Using qPCR

Expression of the XrrA-regulated *inhA1* transcript was assessed using qPCR analysis. The Ames-derived UTA37 (Parent), UTA39 (Δ*xrrA*), UTA39 (pUT1205) (*xrrA* complementation), and the previously published UTA5 (*inhA1*-null) (Pflughoeft et al., 2014) strains were grown in CA-CO_2_ as described above. RNA samples extracted from these strains were treated with 30 units of DNase I (New England Biolabs, Ipswich, MA, United States) for 30 min at 37°C to remove potential DNA contamination. RNA was recovered from the DNase treatment using the RNA Clean and Concentrator Kit (Zymo Research, Irvine, CA, United States). cDNA synthesis was performed using random heptamers and the Super Script III Reverse Transcriptase Kit (Invitrogen, Carlsbad, CA, United States). The resulting cDNA was recovered from the synthesis reaction using the DNA Clean and Concentrator Kit (Zymo Research, Irvine, CA). cDNA samples were subjected to qPCR analysis using *inhA1*-specific primers. The *gyrB* gene, which encodes DNA gyrase subunit B, was used as a reference gene. cDNA and primers were mixed with the SsoAdvanced Universal SYBR Green Supermix (Bio-Rad, Hercules, CA, United States) in duplicate reactions per strain. No reverse transcriptase (NRT) RNA and no template (NTC) water controls were also included. qPCR CT values and melt-curve data were recorded using a CFX96 Real-Time System C1000 Touch Thermal Cycler (Bio-Rad, Hercules, CA, United States). CT values of the duplicate reactions were averaged. The ΔCT values were calculated by subtracting the reference gene *gyrB* CT values from those of *inhA1*. The log_10_ relative expression [2^(–^Δ^CT)^] was calculated for each gene of interest.

### sRNA:mRNA Complementarity and sRNA Secondary Structure *in silico* Analyses

The XrrA and XrrB sequences were entered into the TargetRNA2 webserver (Kery et al., 2014) and aligned to the *B. anthracis* Ames ancestor chromosome (NCBI accession number AE017334.2), pXO1 plasmid (NCBI accession number AE017336.2), and pXO2 plasmid (NCBI accession number AE017335.3) using default parameters. TargetRNA2 takes into account conservation of sRNA sequences, predicted secondary structure of the sRNAs, predicted secondary structure of potential mRNA targets, and the hybridization energy of the sRNA:mRNA interaction and outputs a list of potential targets ranked by hybridization energy, with an adjusted p-value cut-off of 0.05 for each interaction. The TargetRNA2 list of potential targets was compared to the list of sRNA-regulated transcripts obtained from RNA-seq analysis. Transcripts exhibiting a fold-change of ≥4.0 in at least one sRNA-null strain and exhibiting complementarity with the sRNA sequences are reported in Table 4. To determine the most likely predicted secondary structure of XrrA and XrrB, the sRNA sequences were entered into the mfold webserver (Zuker, 2003) and analyzed under default parameters.

### sRNA Half-Life Determinations

The parent strain ANR-1 and the *hfq*-null mutants (Δ*hfq1*, Δ*hfq2*, Δ*hfq3*, Δ*hfq1*Δ*hfq2*Δ*hfq3*) were grown in CA-CO_2_ in triplicate until exponential phase (OD_600_ = 0.6–0.8). After collection of a 750-μl sample (time zero), rifampicin (200 μg ml^–1^) was added to stop transcription. Culture samples were taken at 2, 4, 8, 16, 32, and 45 min post rifampicin addition. Immediately after collection, each sample was mixed with saturated phenol (pH = 4.3) at 65°C and cells were lysed immediately by bead-beating. RNA was extracted from all samples using phenol: chloroform extraction and ethanol precipitation as described above. RNA (3 μg per sample) was subjected to northern blotting, probing for XrrA, XrrB, and 5S rRNA as described above. To validate the half-life determination protocol, the previously known half-life of the *rpsO* transcript, encoding 30S ribosomal protein S15, was determined in the parent strain by probing for *rpsO* using northern blotting. sRNA signal was normalized to 5S rRNA signal per sample for each of the three replicates. sRNA decay over time was calculated as a percent of the signal at time point zero. A linear regression model was fit to the decay data and the slope of the decay lines was used to calculate the half-lives in each replicate per each strain. An analysis of variance (ANOVA) paired with Tukey’s multiple comparison analysis was used to determine significance between the parent strain half-life and each mutant strain half-life, with an adjusted *p*-value cut-off of 0.05.

### Preparation of Vegetative Cells for Mouse Infection

Spores of the ANR-1 strain, the Δ*xrrA* mutant, and the Δ*xrrA*Δ*xrrB* mutant (∼10^7^ CFU) were incubated in 1 ml of BHI at 37°C for 1 h with shaking at 200 r.p.m. The entire outgrowth was transferred into 25 ml of fresh CA medium supplemented with 0.8% sodium bicarbonate. Cultures were incubated in 5% atmospheric CO_2_ at 37°C with shaking at 200 r.p.m. for 4 h. At an OD_600_ of 0.4–0.6, cells were harvested using a 0.22 μm pore filter unit and washed twice with 25 ml Dulbecco’s phosphate-buffered saline without calcium or magnesium (DPBS, Sigma-Aldrich, St. Louis, MO, United States). Cells were finally resuspended in 25 ml of DPBS and diluted to the desired colony forming unit (CFU) concentration, which was verified by plating the inocula on LB agar plates. The inocula were loaded into 1-ml syringes with 27-gauge needles in a final volume of 100 μl.

### Mouse Infections and Organ CFU Determination

All mouse protocols were approved by The University of Texas Health Science Center Institutional Animal Care and Use Committee. Mice were housed in a veterinary-supervised vivarium and had access to unlimited food and water. Seven-week-old female A/J mice, purchased from Jackson Laboratory (Bar Harbor, ME, United States), were sedated prior to infection with 0.1 mg/kg of acepromazine injected intraperitoneally. Mice were infected intravenously via the tail-vein with 100 μl of DPBS containing ∼10^5^ CFU. Fifteen mice were infected with the ANR-1 parent strain, ten mice were infected with the Δ*xrrA* mutant, and nine mice were infected with the Δ*xrrA*Δ*xrrB* mutant. The mice were followed for a period of 11 days and monitored for signs of infection. Moribund mice were sacrificed using CO_2_ asphyxiation, death was verified using cervical dislocation as a secondary method, and time of death was recorded. The liver, lung, spleen, and kidney of sacrificed mice were collected, weighed, and homogenized in 1 ml of DPBS via bead-beating with 2.3-mm diameter zirconia beads (BioSpec Products, Bartlesville, OK, United States). Tissues were homogenized for 1 min, incubated on ice for 1 min, and then homogenized for an additional minute using a Mini Beadbeater (BioSpec Products, Bartlesville, OK, United States). Homogenates were diluted serially and plated on LB agar. Following overnight incubation at 37°C, CFU per gram of tissue was calculated. Survival data were plotted on a Kaplan–Meier curve. An Analysis of Variance (ANOVA), followed by Tukey’s multiple comparisons test was used to calculate significance between CFU organ burden of mice infected with different strains.

## Results

### Transcript Mapping of XrrA and XrrB

We previously investigated regulatory functions of the *B. anthracis* PCVRs, AtxA, AcpA, and AcpB, using RNA-seq to compare gene expression by the Ames parent strain (pXO1^+^, pXO2^+^), an isogenic PCVR-null mutant (Δ*atxA*Δ*acpA*Δ*acpB*), and strains of the PCVR-null mutant complemented with the individual PCVRs (Raynor et al., 2018). While most of the data revealed sequences mapping to annotated loci of the Ames ancestor genome, our RNA-seq read maps showed PCVR-regulated expression of two unannotated loci on the pXO1 virulence plasmid. The apparent high expression level of these RNAs, and the lack of previous annotation suggested that these loci may represent small regulatory RNAs.

**FIGURE 1:**
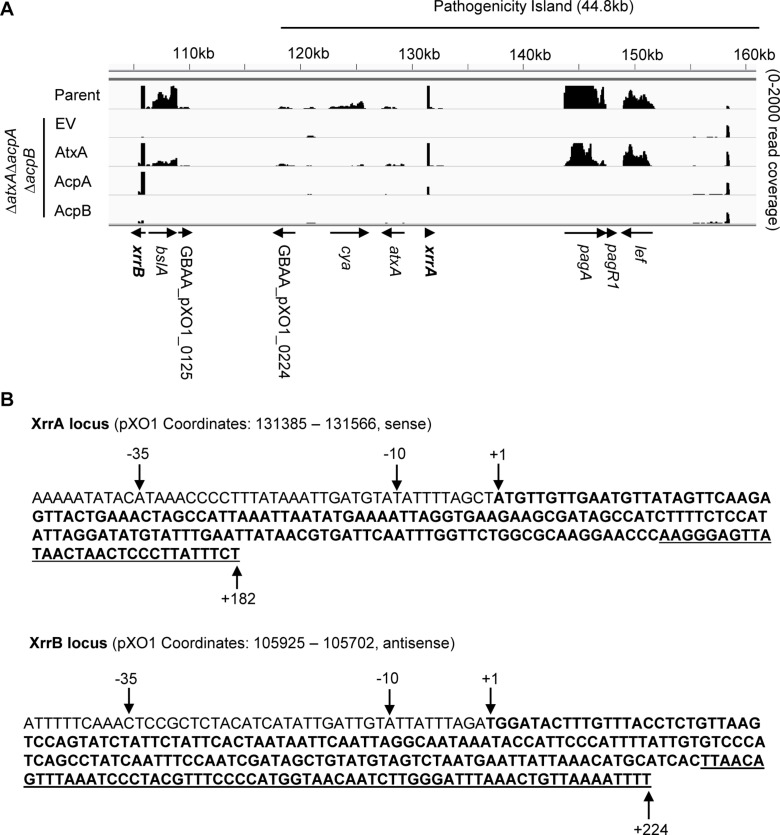
Sequences and PCVR-mediated regulation of sRNA loci on pXO1. **(A)** Read map of the virulence plasmid pXO1, focused on the 44.8 kb pathogenicity island, shows effect of individual PRD-containing virulence regulators (PCVRs) on RNA abundance. RNA-seq data from Raynor et al. (2018) was used to generate read map of gene expression for the Ames parent strain, the Δ*atxA*Δ*acpA*Δ*acpB* strain expressing empty vector (EV), and the individual PCVR complementations in the Δ*atxA*Δ*acpA*Δ*acpB* background. Genes showing apparent regulation by the PCVRs are labeled and direction of transcription is indicated with arrows. XrrA (sense) and XrrB (antisense) are indicated in bold. The boundaries of the pXO1 pathogenicity island, defined by inverted IS1627 elements, are indicated above the read map. **(B)** Sequences and transcriptional direction of the sRNA loci suggested by RNA-seq were confirmed by precise mapping of the 5′ and 3′ ends using RACE. Transcriptional start sites are indicated as +1. The 3′ termini of the transcripts are indicated (+182 for XrrA, +224 for XrrB), and the entire sRNA sequence is shown in bold. Predicted Rho-independent terminator sequences, according to mfold webserver, are underlined. The -10 and -35 nucleotides are shown upstream of the transcriptional start. RACE analysis was repeated 2–3 times per end per sRNA to confirm precise mapping.

To quantify RNAs associated with these loci and verify PCVR-mediated regulation, we re-analyzed raw paired-end reads from Raynor et al. (2018) [raw paired-end reads accessible at NCBI GEO database (Edgar et al., 2002), accession number GSE152357] using the Galaxy web resource for bioinformatic analysis (Afgan et al., 2018) (Figure 1A). We designated the two loci as XrrA and XrrB. In the Ames parent strain, XrrA had 4.5M fragments per kilobase of transcript per million mapped reads (FPKM), while XrrB had 2M FPKM. For comparison, the toxin gene *lef* had 2076 FPKM in the parent strain. XrrA is located within the IS1627 boundaries of the 35-kb pathogenicity island on pXO1, that contains *atxA* and all three anthrax toxin genes. XrrB is located downstream of the pathogenicity island, in proximity to the adhesin gene *bslA*. The Δ*atxA*Δ*acpA*Δ*acpB* mutant, deleted for all PCVRs, exhibited reduced sRNA-associated reads. Expression of both sRNAs was restored upon complementation of the triple-null strain with AtxA, while expression of XrrB was also restored upon complementation with AcpA. The third regulator, AcpB had no effect on XrrA or XrrB expression (Figure 1A).

The RNA sequencing performed by Raynor et al. (2018) was not stranded. We performed Rapid Amplification of cDNA Ends (RACE) experiments to determine the directions of XrrA and XrrB transcription and to precisely map the 5′ and 3′ ends of the sRNAs. Our RACE analysis indicated that XrrA is transcribed from the leading DNA strand, with 5′ and 3′ ends mapping to pXO1 coordinates 131,385 and 131,566, respectively. XrrB is transcribed from the lagging DNA strand of pXO1, with 5′ and 3′ ends mapping to pXO1 coordinates 105,925 and 105,702, respectively. Figure 1B illustrates the loci of the sRNAs with nucleotides corresponding to the 5′ ends of the sRNAs shown as +1. The lengths of the sRNAs as discerned from our RACE analysis are 182 nt for XrrA and 224 nt for XrrB, in agreement with the predicted lengths from the RNA-seq studies.

We used the ORFinder tool from NCBI to search for ATG-initiated open reading frames (ORFs) within the sequences obtained from the RACE analysis. There were no apparent ORFs in XrrB. The XrrA sequence contained a 63 nt ORF with an ATG initiating at pXO1 coordinate 131,488, predicted to encode a 20-amino acid peptide. However, no apparent ribosomal binding site (RBS) could be found upstream of the ORF sequence. Overall, the data indicate that XrrA and XrrB are likely to be non-coding small RNAs.

Small regulatory RNAs may be transcribed from stand-alone promoters to form primary transcripts or result from 5′ or 3′ processing of longer RNA transcripts. Visualization of the 5′ and 3′ RACE PCR products of XrrA and XrrB on an agarose gel showed single bands (Supplementary Figure 1), indicating the formation of a single 5′ and a single 3′ RACE product per sRNA. Moreover, genes adjacent to both ends of the sRNA loci on pXO1 appear to be transcribed in opposite direction from the sRNAs (Figure 1B), making it unlikely that the sRNAs are co-transcribed with other genes. RNA folding predictions using the mFold web server (Zuker, 2003) suggest that both sRNAs form a 3′ hairpin loop followed by a run of uridines (Figure 1B and Supplementary Figures 2, 3), which is indicative of Rho-independent terminators for both XrrA and XrrB. Together, these observations suggest that the sRNAs are primary transcripts.

**FIGURE 2:**
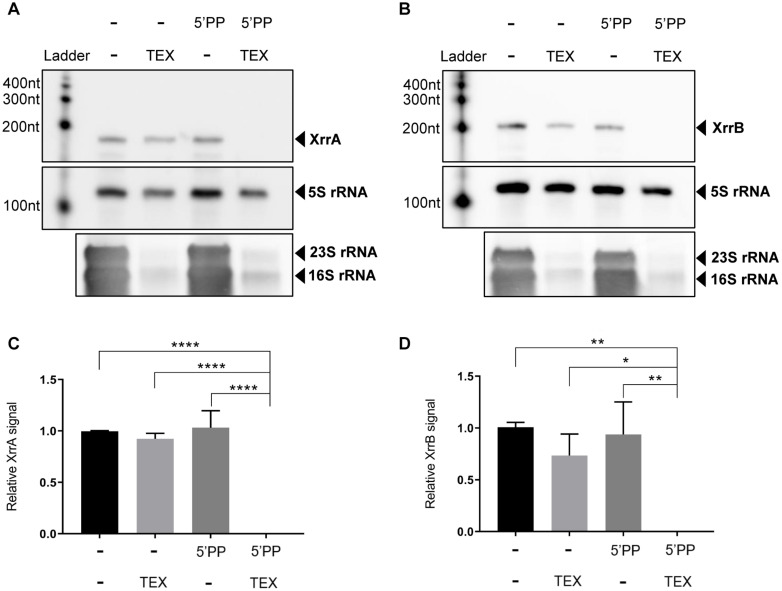
Characterization of XrrA and XrrB 5′ ends. To discern the type of 5′phosphate modification on XrrA and XrrB 5′ ends, total RNA from the parent strain ANR-1 grown in CA-CO_2_ was treated with RNA 5′ Polyphosphatase enzyme (5′PP), and/or Terminator exonuclease enzyme (TEX) in the combinations shown. 5′PP removes the gamma and beta phosphates from primary transcripts with a 5′ triphosphate, leaving a 5′ monophosphate. TEX preferentially degrades transcripts with a 5′ monophosphate. Total RNA was treated, as indicated, followed by northern blotting to probe for **(A)** XrrA and **(B)** XrrB signal. As a positive control for TEX-mediated degradation, levels of 23S and 16S rRNAs were also assessed. The northern blots shown are representative images of three biological replicates. **(C)** XrrA and **(D)** XrrB levels from the three biological replicates were normalized to the TEX-resistant 5S rRNA load control signal and averaged per treatment. The standard deviation in sRNA signal per treatment is shown. Analysis of variance (ANOVA) followed by Tukey’s multiple comparison test was used to determine significance. ^∗^ indicates < 0.05; ^∗∗^ indicates < 0.01; ^****^ indicates < 0.0001.

Primary transcripts in bacteria have a 5′ tri-phosphate group, while secondary transcripts have a 5′ mono-phosphate group. To test whether XrrA and XrrB possess a 5′ tri-phosphate group, we isolated RNA from the ANR-1 strain, treated it with Terminator Exonuclease (TEX), and performed northern blot analysis (Figure 2). The TEX enzyme specifically degrades transcripts with a 5′ monophosphate. XrrA (Figure 2A) and XrrB (Figure 2B) were resistant to TEX-mediated degradation. 5S rRNA was used as a load control and as a negative control for TEX-mediated degradation because it is resistant to degradation by TEX (Patrick et al., 2009; Ferrara et al., 2017; Fleischmann and Rocha, 2018; Lalaouna et al., 2019). As a positive control for TEX-mediated degradation, levels of the TEX-sensitive 16S rRNA and 23S rRNA were assessed. We observed clear degradation of the 16S and 23S rRNAs, but not 5S rRNA, for TEX-treated samples. To confirm that protection of the sRNAs from TEX was due to a tri-phosphate modification at their 5′ ends, we treated RNA with RNA 5′Polyphosphatase (5′PP), which removes the gamma and beta phosphates from the 5′ end of primary transcripts, leaving a 5′ mono-phosphate. Treatment with 5′PP followed by TEX treatment resulted in degradation of both XrrA (Figure 2A) and XrrB (Figure 2B), confirming that protection from TEX was due to the presence of a tri-phosphate at the 5′ ends of the sRNAs. Importantly, treatment with 5′PP alone did not cause sRNA degradation. There was no significant difference in XrrA (Figure 2C) and XrrB (Figure 2D) levels in only TEX-treated and only 5′PP-treated samples. XrrA (Figure 2C) and XrrB (Figure 2D) levels in the 5′PP-TEX-treated samples were significantly reduced compared to all other treatments. These data indicate that XrrA and XrrB are primary transcripts originating from stand-alone promoters.

**FIGURE 3:**
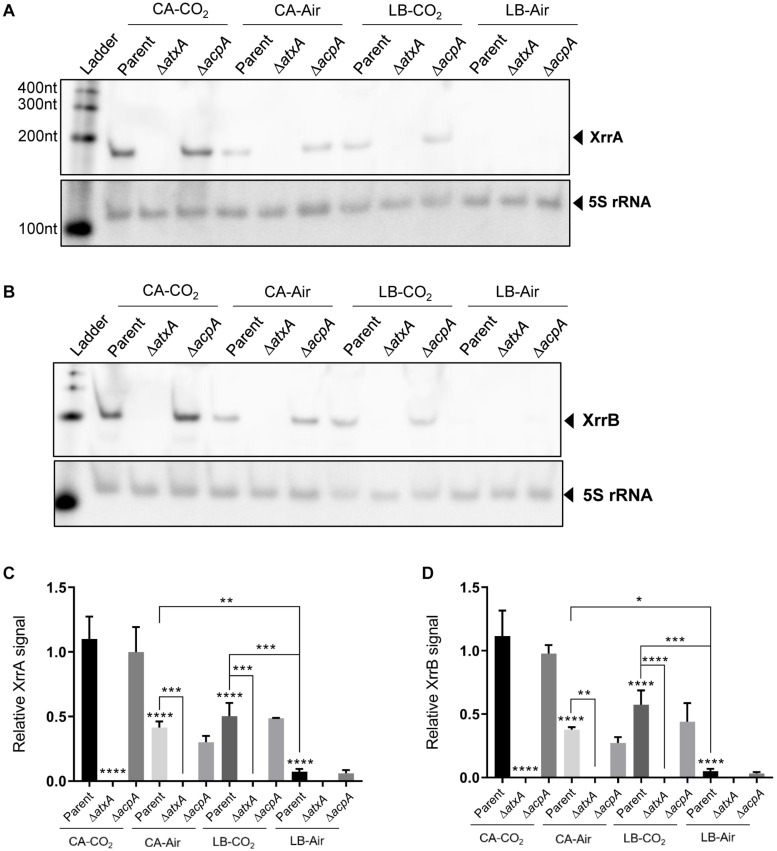
AtxA-mediated control of XrrA and XrrB, and influence of growth conditions that affect AtxA expression and activity on sRNA expression. Total RNA from the Ames parent strain, the Δ*atxA* mutant, and the Δ*acpA* mutant was extracted from cultures grown in the indicated conditions until early stationary phase (OD_600_ = 1.0–1.5). Cultures were grown in CA or LB medium and exposed to 5% atmospheric CO_2_ or air during growth. Expression of **(A)** XrrA and **(B)** XrrB was assessed using northern blotting, and the 5S rRNA signal was used as a load control. A representative northern blot from three biological replicates is shown. **(C)** XrrA and **(D)** XrrB levels from the three biological replicates were normalized by 5S rRNA and averaged. The standard deviation in sRNA signal per sample is shown. Analysis of variance (ANOVA) followed by Tukey’s multiple comparison test was used to determine significance. ^∗^ indicates < 0.05; ^∗∗^ indicates < 0.01; ^∗∗∗^ indicates < 0.001; ^****^ indicates < 0.0001. Asterisks directly above bars indicate significance of comparison between that bar and the “Parent CA-CO_2_” condition. Additional comparisons between conditions are shown linked by brackets, with respective significance indicated by asterisks above the brackets.

### sRNA Expression in Cultures Grown in Conditions That Influence AtxA Expression and Activity

Data from previous reports indicate that XrrA and XrrB transcript levels are positively regulated by AtxA (McKenzie et al., 2014; Raynor et al., 2018). To further explore AtxA-mediated control of XrrA and XrrB, we asked whether sRNA expression patterns are influenced during culture conditions that affect AtxA expression and activity. Transcript levels of *atxA* in cultures grown in minimal media containing glucose are higher than levels in cultures grown in rich media (Chiang et al., 2011). Several reports have described a positive effect of CO_2_/bicarbonate on anthrax toxin production (Koehler et al., 1994; Dai et al., 1995; Hammerstrom et al., 2011). Elevated CO_2_/bicarbonate levels in media enhance dimerization of AtxA (Hammerstrom et al., 2011), which is required for its activity (Hammerstrom et al., 2015).

We cultured cells at 37°C in the semi-defined minimal medium CA, which contains 0.1% w/v of glucose, or the rich complex medium LB with no added glucose. CA and LB cultures were incubated shaking in air (CA-Air, LB-Air) or in 5% CO_2_ (with 0.8% sodium bicarbonate added to the medium) (CA-CO_2_, LB-CO_2_). sRNA expression was assessed in the Ames parent strain using northern blotting (Figure 3). Representative northern blots are shown in Figure 3A (XrrA) and Figure 3B (XrrB). Averages of three biological replicates are represented in Figure 3C (XrrA) and Figure 3D (XrrB). Overall, expression patterns for the two sRNAs were similar. sRNA levels were elevated when cultures were incubated in CA medium, relative to LB, and when media were supplemented with 0.8% sodium bicarbonate and incubated in 5% atmospheric CO_2_, relative to media lacking the sodium bicarbonate supplement and incubated in air. XrrA and XrrB levels were 16- and 22-fold higher in cultures grown in CA-CO_2_ compared to LB-Air, respectively. When cultured in air, XrrA and XrrB levels were 7.0-fold greater in CA compared to LB. The CO_2_ effect was apparent in both media. XrrA and XrrB levels were 3.0-fold greater in CA-CO_2_ compared to CA-Air. XrrA and XrrB levels were elevated 7.0- and 11-fold, respectively, in LB-CO_2_ compared to LB-Air. Finally, for both sRNAs, expression levels were 2.0-fold higher in cultures grown in CA-CO_2_ compared to cultures grown in LB-CO_2_.

Deletion of *atxA* resulted in no detectable sRNA expression in all the conditions tested (Figure 3). Notably, although previously reported RNA-seq data suggested that AcpA positively affected XrrB expression (Raynor et al., 2018), in our experiments deletion of *acpA* did not alter XrrB levels compared to the parent strain. In the previous study (Raynor et al., 2018), AcpA control of XrrB was observed when an Δ*atxA*Δ*acpA*Δ*acpB* strain was complemented with AcpA. Our studies using an *acpA*-null mutant carrying the native *atxA* gene suggest that XrrB is responsive to AtxA in the absence of AcpA. Together, the data indicate that XrrA and XrrB are primarily regulated by AtxA, and that sRNA expression patterns mimic the *atxA*-dependent expression of *B. anthracis* virulence factors such as the anthrax toxin genes.

### sRNA Half-Life in Parent and hfq-Null Strains

In some bacteria, sRNAs are stabilized by the RNA-chaperone Hfq. Interactions with Hfq lead to protection against RNases and facilitate base-pairing with mRNA targets. The *B. anthracis* genome includes three genes predicted to encode Hfq proteins. Two of these proteins, Hfq1 and Hfq2, are encoded on the chromosome, while a third protein, Hfq3, is encoded on pXO1 (Vrentas et al., 2015). Positive control of the pXO1-encoded sRNAs and the chromosome-encoded Hfq2 by AtxA (McKenzie et al., 2014; Raynor et al., 2018), and the previously reported functionality of Hfq2 and Hfq3 in *E. coli* (Vrentas et al., 2015; Keefer et al., 2017), indicate potential relationships between the sRNAs and Hfq proteins of *B. anthracis*.

**FIGURE 4:**
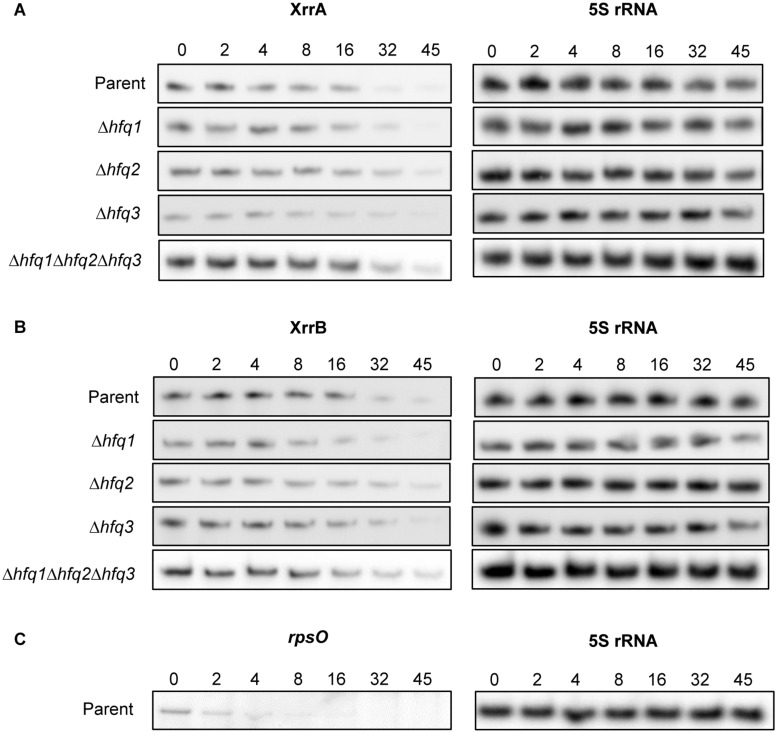
Half-life determination experiments using rifampicin treatment of growing cells. The ANR-1 parent strain, the Δ*hfq1* mutant, the Δ*hfq2* mutant, the Δ*hfq3* mutant, and the Δ*hfq1*Δ*hfq2*Δ*hfq3* mutant were grown in CA-CO_2_ until late exponential phase (OD_600_ = 0.6–0.8), followed by addition of 200 μg/ml of rifampicin to stop transcription initiation. Culture samples were taken immediately before rifampicin addition (indicated by time point 0) and 2, 4, 8, 16, 32, and 45 min post-addition of rifampicin. RNA was extracted from culture samples and subjected to northern blotting to assess **(A)** XrrA and **(B)** XrrB decay over time and calculate half-lives. **(C)** The half-life of the *rpsO* transcript, encoding the 30S ribosomal protein S15, in the parent strain was also calculated as an experimental control. RNA decay was calculated as the fraction of RNA signal normalized to 5S rRNA at time point 0. A linear regression was fit to averaged data from three biological replicates to calculate the slope of the decay, which was then used to calculate the half-lives. Analysis of variance (ANOVA) followed by Tukey’s multiple comparison analysis was used to determine significance in half-life differences between strains. Representative northern blots from the three biological replicates are shown.

A common feature of Hfq-dependent sRNAs is the presence of Rho-independent terminators at the 3′ ends of the transcripts (Otaka et al., 2011). The 3′ oligoU tails of these sRNAs are often bound by Hfq (Sauer and Weichenrieder, 2011; Ishikawa et al., 2012). Given that XrrA and XrrB are predicted to form Rho-independent terminators (Supplementary Figures 2, 3), we asked whether the *B. anthracis* Hfq chaperones influenced XrrA and XrrB stability. We constructed isogenic deletion mutants for the *hfq* genes (Δ*hfq1*,Δ*hfq2*,Δ*hfq3, and*Δ*hfq1*Δ*hfq2*Δ*hfq3*) and measured the sRNA half-lives in parent and mutant strains when cultured in toxin-inducing conditions (Figure 4). XrrA and XrrB had similar half-lives in the parent strain. The half-life of XrrA was 20 ± 1.1 min (Figure 4A and Table 2), while the half-life of XrrB was 21 ± 1.5 min (Figure 4B and Table 2). Deletion of the *B. anthracis hfq* genes had no statistically significant effects on sRNA stability, although there was a slight trend toward extension of sRNA half-life in some mutants. To validate our RNA stability assay, we tested the stability of the *rpsO* transcript, which encodes a ribosomal protein of the small sub-unit of the ribosome. The *B. subtilis rpsO* transcript has been reported to be approximately 4 min (Yao and Bechhofer, 2010). In our experimental conditions, the half-life of the *B. anthracis rpsO* transcript was 2.1 ± 1.2 min (Figure 4C and Table 2). Overall, these data indicate that when *B. anthracis* is cultured in optimal conditions for toxin gene expression, XrrA and XrrB are highly stable, Hfq-independent sRNAs.

**TABLE 2:** sRNA half-lives in the parent and *hfq*-null strains.

Strain	XrrA Half-life (minutes)^a^	XrrB Half-life (minutes)^a^	*rpsO* Half-life (minutes)^a^
Parent	20 ± 1.1	21.2 ± 1.5	2.1 ± 1.2
Δ*hfq1*	20.4 ± 0.9	20.2 ± 0.2	
Δ*hfq2*	25.1 ± 4.4	23 ± 3.4	
Δ*hfq3*	24.7 ± 4.6	21.4 ± 3.4	
Δ*hfq1*Δ*hfq2*Δ*hfq3*	23 ± 1.6	25.2 ± 2.3	

*^*a*^Half-lives reported are the average of three biological replicates, shown in minutes.*

*Standard deviation in sRNA and rpsO mRNA half-lives is indicated as ±minutes*.

### sRNA Regulons and Loci of sRNA-Regulated Genes

To determine sRNA-controlled genes of *B. anthracis*, we compared the transcriptomes of *xrrA*- and *xrrB*-null mutants to that of a parent strain using RNA-seq. We constructed sRNA-null mutants in a virulent Ames (pXO1^+^, pXO2^+^) background. We compared the transcriptome of UTA37 (Raynor et al., 2018) with that of the isogenic mutants UTA38 (Δ*xrrB*), UTA39 (Δ*xrrA*), and UTA41 (Δ*xrrA*Δ*xrrB*). Cultures were grown to early stationary phase in CA-CO_2_, which allows high level expression of the sRNAs (Figure 3). RNA was extracted and subjected to Illumina sequencing.

**FIGURE 5:**
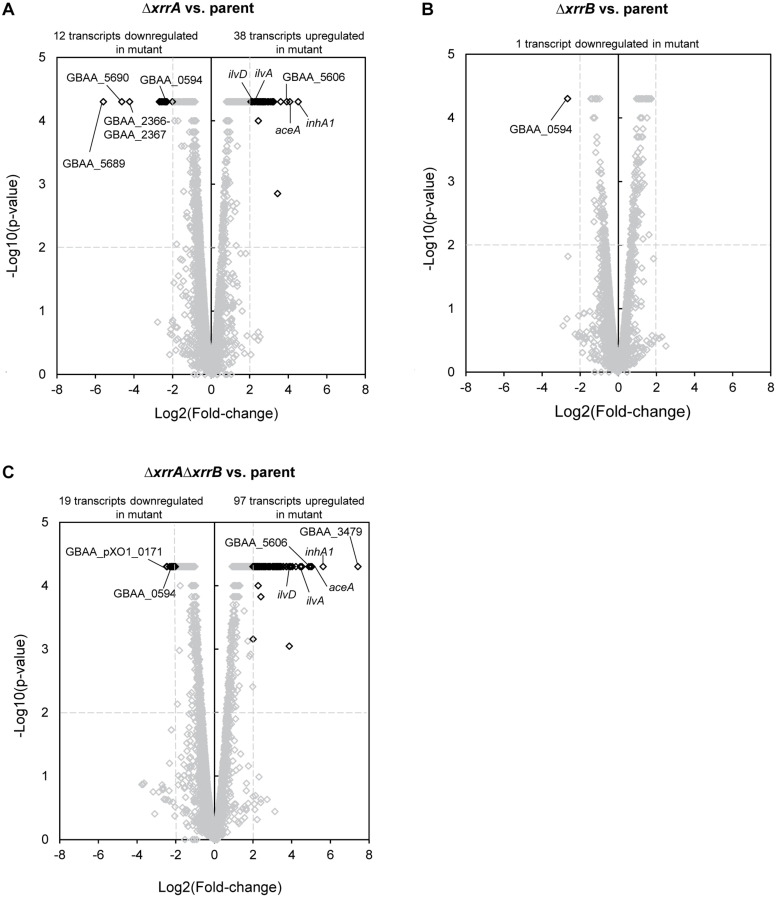
Transcriptomic analysis of sRNA-null mutants compared to the parent strain. The Ames-derived UTA37 parent strain, UTA38 (Δ*xrrB*), UTA39 (Δ*xrrA*), and UTA41 (Δ*xrrA*Δ*xrrB*) were grown in CA-CO_2_ to early stationary phase (OD_600_ = 1.0–1.5) and RNA was extracted for RNA-seq analysis. Volcano plots show the effect of **(A)** Δ*xrrA* deletion, **(B)** Δ*xrrB* deletion, and **(C)** Δ*xrrA*Δ*xrrB* deletion on RNA abundance, compared to the parent strain. Transcripts that showed significantly different expression levels are highlighted in black. Transcripts that did not show a significant difference in expression are shown in gray. The *p*-value cutoff was 0.01, which corresponds to a –log10(*p*-value) of 2, and the log2(fold-change) cutoff was ≥2.0, which represents a fold-change of ≥4.0. Significance and fold-change thresholds are indicated by dashed lines. Transcripts of interest are labeled.

Deletion of *xrrA* and *xrrB* had distinct effects on *B. anthracis* gene expression (Figure 5). Deletion of *xrrA* resulted in 50 transcripts showing a ≥4.0-fold change in expression compared to the parent strain; expression of 12 transcripts was reduced in the Δ*xrrA* mutant, while 38 transcripts were elevated in the mutant (Figure 5A). In contrast, deletion of *xrrB* led to one transcript having a ≥4.0-fold change in expression compared to the parent strain (Figure 5B). The transcript, encoded by the gene GBAA_0594, was decreased in the Δ*xrrB* mutant. Interestingly, deletion of both sRNAs in the Δ*xrrA*Δ*xrrB* mutant affected a greater number of transcripts than the combined total number of transcripts affected in the Δ*xrrA* and Δ*xrrB* mutants. In the Δ*xrrA*Δ*xrrB* mutant, levels of 116 transcripts were affected with a fold-change of ≥4.0 (Figure 5C). Ninety-seven of these transcripts were increased in the mutant, while 19 transcripts were decreased. Our analysis detected one apparent operon and nine bicistronic transcripts amongst all transcripts affected by sRNA deletions (Supplementary Table 2). The apparent operon (*hom1-thrC-thrB*) encodes enzymes involved in threonine biosynthesis and its expression was elevated in the Δ*xrrA* and Δ*xrrA*Δ*xrrB* mutants (Supplementary Table 2). Eight of the nine bicistronic transcripts were elevated in sRNA mutant strains. One bicistronic transcript (GBAA_2366-GBAA_2367) was decreased in the Δ*xrrA* and Δ*xrrA*Δ*xrrB* mutants (Supplementary Table 2).

Most sRNA-regulated genes uncovered in this study were located on the *B. anthracis* chromosome. Of all transcripts exhibiting a fold-change of ≥4.0, only four were associated with genes on pXO1. Three of these genes encode hypothetical proteins with no ascribed functions (Supplementary Table 2). These transcripts were encoded by the GBAA_pXO1_0022, GBAA_pXO1_0153, and GBAA_pXO1_0171 genes. GBAA_pXO1_0022 was the only affected pXO1 gene not located within the plasmid pathogenicity island. Expression of all three of these genes was affected in both the Δ*xrrA* and Δ*xrrA*Δ*xrrB* strains. The fourth sRNA-regulated pXO1 gene was *lef*, encoding the lethal factor component of the anthrax toxin, located within the pXO1 pathogenicity island. Interestingly, expression of the *lef* transcript was reduced 4.7-fold in the Δ*xrrA*Δ*xrrB* mutant and unaffected in the single deletion strains. Finally, there were no pXO2-encoded transcripts regulated by the sRNAs having a fold-change of ≥4.0 compared to the parent.

### Overlap Between sRNA Regulons

Table 3 lists transcripts that were most highly-regulated (≥16-fold-change) by the individual sRNAs and transcripts that were regulated by both sRNAs. Supplementary Table 2 is an expanded version of Table 3, listing transcripts that were regulated ≥4.0-fold by individual sRNAs and transcripts regulated by both sRNAs. The transcript encoded by GBAA_0594 was the only transcript affected in all sRNA deletion mutants with a ≥4.0 fold-change (Figure 5 and Supplementary Table 2). GBAA_0594 was also the sole transcript that showed a fold-change of ≥4.0 in the Δ*xrrB* mutant (Figure 5B).

**TABLE 3:** Transcripts most highly regulated and co-regulated by the sRNAs.

			Log2(fold-change)^a^		
	
Transcript tag	Gene name(s)	Function	Δ*xrrA*	Δ*xrrB*	Δ*xrrA*Δ*xrrB*
GBAA_3479		Putative ArsR-family transcriptional regulator	–	–	+7.43
GBAA_1295	*inhA1*	Immune inhibitor metalloprotease	+4.52	+1.02	+5.63
GBAA_1132	*aceA*	Isocitrate lyase	+4.08	–	+5.05
GBAA_5606		Putative aminopeptidase	+3.92	–	+4.99
GBAA_2827		Putative chitin binding protein	+3.21	+0.98	+4.89
GBAA_1854	*ilvA*	Threonine ammonia-lyase	+2.56	–	+4.53
GBAA_4149		Putative hydrolase	+2.62	–	+4.44
GBAA_2633		Putative cysteine deoxygenase	+2.06	–	+4.44
GBAA_3709-GBAA_3710	*hutG-hutI*	Formiminoglutamase-imidazolonepropionase	+3.21	–	+4.22
GBAA_1853	*ilvD*	Dihydroxy-acid dehydratase	+2.26	–	+4.04
GBAA_2366- GBAA_2367		Hypothetical protein-putative oxalate:formate antiporter	−4.23	–	−1.48
GBAA_5690		Putative holin	−4.63	–	−1.2
GBAA_5689		Putative membrane protein	−5.6	–	−1.6

**^*a*^Hyphens indicate a non-significant difference in transcript expression*.*

**TABLE 4:** Regions of complementarity between the sRNAs and sRNA-regulated mRNA transcripts.

sRNA sequence^a^	sRNA start^b^	sRNA end^b^	mRNA^c^	mRNA sequence^a,d^	mRNA start^e^	mRNA end^e^	Energy^f^	*p*-value^g^
**XrrA**								
**Seed region # 1**								
aGUCAUUGAGAAcUUGAu	34	17	*asnO2*	aUAGUAACUCUUuAACU	−72	−56	−11.97	0.008
**Seed region # 2**								
CCUCu UUUCUACCG-AUagCGa	92	72	GBAA_4468	GGAG-GGAGAUGGC**aUG**gaGC	−14	+7	−13.21	0.003
uAUACCUCUUUUCUaCCg	96	79	GBAA_5301	uUAUGGAGAAAAGGaGG	−27	−11	−14.83	0.001
aUACCUCUUUucUACc	95	80	GBAA_0656	gGUGGAGAAGuu**AUG**a	−12	+4	−11.58	0.01
**Seed region # 3**								
aUUCCCUCAAUcAAUAUu	177	160	GBAA_3451	aGGGGGAGUUA-UUAU**Au**	−15	+2	−14.81	0.001
uCUUUaUUCCCUCa	182	169	*hutU*	uGAGAaAAGGGAGa	−21	−8	−13.72	0.002
U-CUUUAUUCCCUc	182	170	*inha1*	AgGAAAUAAGGG**Au**	−12	+2	−11.54	0.01
**XrrB**								
AUGGUaCCCCUUUg	194	180	GBAA_2549	UACUAaGGGGGAAa	−19	−6	−13.7	0.002

*^*a*^Complementarity between sequences is shown in uppercase letters. Dashes indicate a break in the complementarity region.*

*^*b*^Indicates start and end of complementarity region on the sRNA, relative to the sRNA transcriptional start site.*

***^*c*^**Transcripts exhibiting a fold-change of ≥4.0 in at least one sRNA-null strain were selected for analysis.*

*^*d*^Nucleotides that are part of the translational start codon of the mRNA are shown in bold.*

*^*e*^Indicates start and end of complementarity region on the mRNA, relative to the mRNA translational start site.*

*^*f*^Thermodynamic energy (kcal/mol) of hybridization between sRNA and mRNA, as calculated by TargetRNA2.*

*^*g*^Indicates probability that the sRNA and mRNA interaction occurs by chance (p-value cutoff was 0.05), as calculated by TargetRNA2*.

**FIGURE 6:**
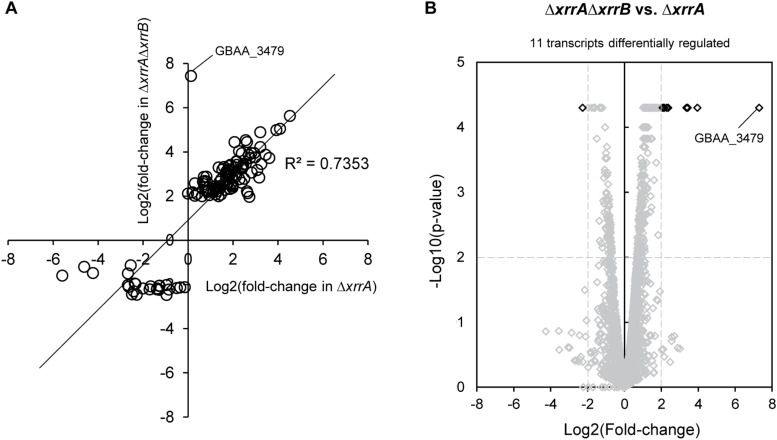
Comparison of sRNA regulons uncovered by RNA-seq analysis. **(A)** Scatterplot graph showing the log2(fold-change) differences in transcript expression compared to the parent strain in the Δ*xrrA* and Δ*xrrA*Δ*xrrB* mutants. Log2(fold-change) values from the Δ*xrrA* and Δ*xrrA*Δ*xrrB* mutants are plotted on the *x*- and *y*-axis, respectively. Transcripts with a significant log2(fold-change) of ≥2.0 in at least one mutant strain are shown as circles. A linear regression model was fit to the data, and the *R*^2^ is shown. **(B)** Volcano plot showing a direct comparison of transcripts differentially regulated between the Δ*xrrA* and Δ*xrrA*Δ*xrrB* mutants. Transcripts that were significantly differentially expressed between the deletion strains are highlighted in black. Transcripts that were not differentially regulated are shown in gray. The *p*-value cutoff was 0.01, which corresponds to a –log10(*p*-value) of 2, and the log2(fold-change) cutoff was ≥2.0, representing a fold-change of ≥ 4.0. Significance and fold-change thresholds are indicated by dashed lines.

Given that XrrA regulated many more transcripts than XrrB, and that deletion of both *xrrA* and *xrrB* resulted in even further changes to transcript expression, we compared the XrrA and XrrAXrrB regulons obtained from RNA-seq analysis (Figure 6). To visualize overlap between sRNA regulons, we plotted transcripts exhibiting a significant fold-change of ≥4.0 in at least one mutant strain as a scatterplot (Figure 6A). We found that most transcripts affected in the Δ*xrrA*Δ*xrrB* mutant were also affected in the Δ*xrrA* mutant. All transcripts were regulated in the same direction, either increased or decreased expression, in both strains (Figure 6A). In addition, for most of the regulated genes, the fold-changes in the Δ*xrrA* mutant and the Δ*xrrA*Δ*xrrB* mutant were comparable. The data indicate that for most transcripts, regulatory effects observed in the double mutant likely result from deletion of *xrrA*. Nevertheless, we observed some transcripts that exhibited non-significant changes in expression in the Δ*xrrA* mutant but were significantly altered in the Δ*xrrA*Δ*xrrB* mutant. To confirm that these transcripts were affected only in the double null mutant, we directly compared transcript expression in the Δ*xrrA* and Δ*xrrA*Δ*xrrB* mutants (Figure 6B). Indeed, we found 11 transcripts for which expression was affected ≥4.0-fold in the Δ*xrrA*Δ*xrrB* mutant but were not affected ≥4.0-fold in the Δ*xrrA* mutant. Together, the data suggest distinct regulatory roles for XrrA, as well as some functional overlap between XrrA and XrrB, as evidenced by the increased number of XrrB-regulated targets in the *xrrA-xrrB*-null background.

Notably, our data show that the sRNAs do not regulate expression of each other or *atxA*. Northern blot analysis of XrrA and XrrB in sRNA-null strains showed comparable levels of XrrA expression in the parent and Δ*xrrB* mutant (Supplementary Figure 4). Similarly, XrrB levels were comparable in the parent and Δ*xrrA* mutant (Supplementary Figure 4).

### Most-Highly sRNA-Regulated Targets

RNA-seq data uncovered GBAA_0594 as the only transcript controlled by XrrB in the presence of XrrA (Figure 5B). GBAA_0594 is predicted to encode a putative transcriptional regulator of the ArsR-family. These regulators typically bind DNA in the presence of metal cofactors (Ren et al., 2017). Expression of GBAA_0594 was reduced in the Δ*xrrB* mutant compared to the parent strain, with a fold-change of 6.3 (Figure 5B and Supplementary Table 2). This transcript was also decreased in the Δ*xrrA* and Δ*xrrA*Δ*xrrB* mutants, with fold-changes of 5.7 and 5.4, respectively (Figure 5 and Supplementary Table 2). Decreased levels of GBAA_0594 in the *xrrB*-null did not result in changes in expression of other transcripts in this mutant, suggesting that the encoded protein may not function as a transcriptional regulator, at least in the growth conditions tested.

Overall, XrrA regulated many more genes than XrrB. One of the most highly XrrA-regulated transcripts is encoded by the *inhA1* gene (Figure 5A). InhA1 is a secreted protease that mediates processing of *B. anthracis* proteins and break-down of host proteins during infection (Pflughoeft et al., 2014; Terwilliger et al., 2015). Expression of *inhA1* was elevated 23-fold in the Δ*xrrA* mutant compared to the parent strain (Table 3), suggesting that XrrA represses expression of *inhA1*. The transcript was also differentially regulated in the Δ*xrrA*Δ*xrrB*, with a fold-change of 50 (Table 3). Deletion of XrrB had a 2.0-fold effect on *inhA1* expression, indicating a synergistic effect of the sRNAs on *inhA1* expression.

XrrA also appears to regulate enzymes of the glyoxylate cycle. This cycle is a variation of the tricarboxylic acid (TCA) cycle that allows some organisms, including bacteria, to bypass decarboxylation steps of the TCA cycle to synthesize succinate from acetyl-coA (Kornberg and Madsen, 1989). The two enzymes, isocitrate lyase and malate synthase, are encoded by the *aceA* and *aceB* genes, respectively. Expression of *aceA* was increased in the Δ*xrrA* and Δ*xrrA*Δ*xrrB* mutants, with fold-changesx of 17 and 33, respectively (Figure 5 and Table 3). Expression of *aceB* was elevated 7.9- and 13-fold in the Δ*xrrA* and Δ*xrrA*Δ*xrrB* mutants (Supplementary Table 2).

Deletion of *xrrA* also affected genes predicted to be involved in branched-chain amino acid (BCAA) biosynthesis, transport, and catabolism. Previous reports have indicated AtxA-mediated repression of BCAA biosynthesis operons and BCAA transporter genes (Bourgogne et al., 2003; Raynor et al., 2018). According to our own RNA-seq data, the two most highly sRNA-regulated BCAA-related genes were *ilvA* and *ilvD*, which encode enzymes of the BCAA biosynthesis pathway (Figure 5 and Table 3). The *ilvA* gene encodes an enzyme that catalyzes the first step of isoleucine biosynthesis from threonine, while *ilvD* encodes an enzyme utilized in the biosynthesis of all three BCAAs. The *ilvA* transcript increased 5.9-fold in the Δ*xrrA* mutant (Figure 5A and Table 3). Interestingly, in the Δ*xrrA*Δ*xrrB* mutant the *ilvA* transcript increased 23-fold compared to the parent (Figure 5C and Table 3). The *ilvD* transcript displayed similar effects, with the double sRNA deletion having a greater effect on expression than the single *xrrA* deletion (Figure 5 and Table 3). Interestingly, deletion of *xrrB* alone did not affect *ilvA* or *ilvD* expression. XrrA also regulated other enzymes of the BCAA biosynthesis operons of *B. anthracis*, as well as enzymes involved in threonine biosynthesis (*hom1-thrC-thrB*) (Supplementary Table 2). Threonine serves as a precursor for isoleucine biosynthesis. At least one gene predicted to be involved in BCAA transport, *brnQ3*, was regulated by XrrA. Based on sequence conservation, BrnQ3 is predicted to function as a sodium-dependent transmembrane transporter of BCAAs. The transcript was differentially regulated 3.7-fold in the Δ*xrrA* mutant, and 9.1-fold in the Δ*xrrA*Δ*xrrB* mutant (Supplementary Table 2). Expression of genes encoding enzymes involved in BCAA catabolism was also impacted. Transcript levels of the *bfmbAa*, *bfmbB*, and *bfmbAb* genes, encoding components of the branched-chain alpha-keto dehydrogenase complex, were reduced in the Δ*xrrA* mutant, suggesting that XrrA positively influences expression of BCAA catabolism while at the same time repressing BCAA biosynthesis and transport (Supplementary Table 2).

The most highly sRNA-regulated transcript in *B. anthracis* according to our RNA-seq analysis was GBAA_3479, which encodes a second putative ArsR-family transcriptional regulator. Interestingly, expression of GBAA_3479 was only affected in the Δ*xrrA*Δ*xrrB* mutant (Figure 6 and Table 3). Expression of GBAA_3479 was increased 172-fold in this mutant. Overall, the data indicate that XrrA primarily functions to repress expression of targets, and that at least one target requires both XrrA and XrrB for regulation.

Given that *inhA1* was the most-highly regulated target controlled by a single sRNA, we sought to confirm XrrA-mediated regulation of *inhA1*. We used qPCR to compare relative *inhA1* levels in the Ames-derived parent strain UTA37, the Δ*xrrA* mutant UTA39, and UTA39 complemented with pUT1205 containing *xrrA* under the control of an IPTG-inducible promoter. The *inhA1*-null (*inhA1::specR*) mutant UTA5 was used as control for primer specificity. In agreement with our RNA-seq analysis, the qPCR data (Supplementary Figure 5) showed that *inhA1* expression was elevated 10- to 15-fold in the *xrrA*-null mutant compared to the parent. Moreover, exogenous complementation of *xrrA* lowered *inhA*1 expression to levels similar to the parent strain. These data confirm XrrA-mediated regulation of *inhA1* and validate our RNA-seq data.

### Gene Ontology Analysis of sRNA Regulons

**FIGURE 7:**
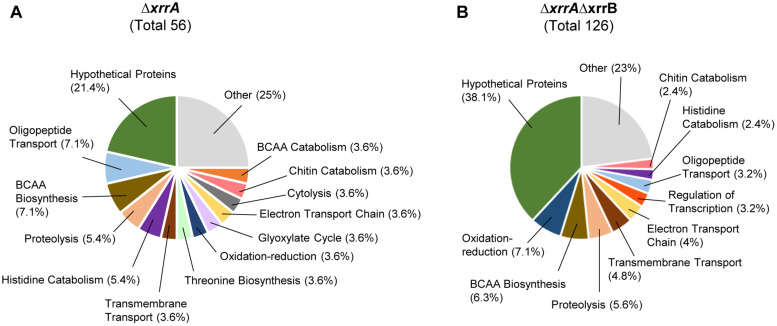
Gene ontology analysis of genes of which expression was affected in the Δ*xrrA* and Δ*xrrA*Δ*xrrB* strains. Genes that were differentially regulated in the **(A)** Δ*xrrA* and **(B)** Δ*xrrA*Δ*xrrB* mutants with a fold-change of ≥4.0 compared to the parent strain were categorized based on biological processes. Pie charts show the total number of differentially regulated genes and the percentage of those genes that belong to a gene ontology category based on biological processes. Hypothetical proteins represent genes with unknown functions or no putative functions based on gene sequence analysis. The colored sections of the pie charts represent biological process categories associated with more than 2% of the strain regulons. Categories associated with 2% or less of the regulons are shown in gray and labeled as “other.”

We performed gene ontology analysis of genes regulated by the sRNAs. Since XrrB regulated a single target, we chose to focus on classification of genes affected ≥4.0-fold in the Δ*xrrA* and Δ*xrrA*Δ*xrrB* mutants according to their predicted biological function (Figure 7). Hypothetical proteins of unknown function comprised 21% of the XrrA regulon (Figure 7A). Interestingly, genes encoding proteins predicted to be involved in oligopeptide transport represented approximately 7% of the XrrA regulon. These included GBAA_0656, GBAA_0658, and GBAA_0852, which are predicted to be ABC-type transporters (Supplementary Table 2). Transcripts from these genes appear to be repressed by XrrA. BCAA biosynthesis genes represented an additional 7% of the XrrA regulon. About 5% of the XrrA-affected genes are involved in proteolysis (Figure 7A). These included *inhA1*, GBAA_5606, and *calY*. The *calY* gene encodes camelysin, a cell surface-bound metalloprotease involved in virulence in *B. cereus* (Grass et al., 2004; Candela et al., 2019). An additional 5% of the XrrA regulon consists of genes associated with histidine catabolism, including genes of the *histidine utilization* (*hut*) operon. The *hutG-hutI* transcript was regulated 9.3-fold in the Δ*xrrA* strain (Table 3) and the *hutU* transcript was regulated 5.4-fold in this strain (Supplementary Table 2).

For genes whose expression was altered in the Δ*xrrA*Δ*xrrB* mutant, 38% were genes encoding hypothetical proteins with no ascribed functions (Figure 7B). Interestingly, approximately 7% of the genes of the XrrAXrrB regulon encode proteins that mediate oxidation-reduction reactions (Figure 7B). These included genes encoding ubiquinone, menaquinol, and putative cytochromes (Supplementary Table 2). BCAA biosynthesis genes represented 6.3% of the XrrAXrrB regulon, and proteolysis genes represented 5.6%. Regulation of transcription was a biological process represented only in the XrrAXrrB regulon (3.2%), in part given by control of the putative transcriptional regulator GBAA_3479 in the Δ*xrrA*Δ*xrrB* mutant only. Other represented biological processes included histidine catabolism, transmembrane transport, and chitin catabolism.

### *In silico* Analysis of Complementarity Between the sRNAs and mRNA Targets

A common mechanism of sRNA function is direct base-pairing with mRNA targets to control aspects of translation and/or transcript decay. Base-pairing to the mRNA target often occurs via a short, imperfect region of complementarity often referred to as the seed region (Gottesman and Storz, 2011). We asked whether the sRNAs displayed any complementarity to sRNA-regulated mRNA transcripts uncovered in this study. To find potential interactions between the sRNAs and mRNAs, we used the TargetRNA2 webserver (Kery et al., 2014). sRNA sequences uncovered by RACE analysis were entered to the TargetRNA2 webserver and aligned to the *B. anthracis* chromosome. Transcripts displaying a fold-change of ≥4.0 in at least one sRNA-null strain and showing complementarity to the sRNAs are listed in Table 4. We found seven XrrA-regulated transcripts that had complementarity to XrrA. For four of the transcripts, XrrA was predicted to interact at the translational start site of the mRNA, suggesting that XrrA may influence translation of these transcripts. The transcripts *inhA1*, GBAA_0656, and GBAA_4468 are negatively regulated by XrrA (Table 4 and Supplementary Table 2). Thus base-pairing with XrrA may result in inhibition of translation, leading to mRNA decay due to reduced ribosomal occupancy. On the other hand, GBAA_3451 is positively regulated by XrrA (Supplementary Table 2), suggesting that the base-pairing interaction may result in enhanced translation of the transcript. For the remaining 3 transcripts, XrrA was predicted to interact further upstream in the 5′ UTR. For *hutU*, which encodes urocanate hydratase and is negatively regulated by XrrA (Supplementary Table 2), base-pairing with XrrA is predicted to occur eight nucleotides upstream of the translational start site, suggesting that XrrA may block the RBS of *hutU* to inhibit translation. The remaining two transcripts, GBAA_5301 and *asnO2*, are predicted to base-pair with XrrA further upstream in their respective 5′UTRs. Such interactions could affect secondary structure and stability of the transcripts, resulting in changes to the rate of mRNA decay. Overall, our data suggest that XrrA is likely to function as a base-pairing sRNA.

Interestingly, the seven mRNA transcripts that showed complementarity to XrrA were all targeted by one of three seed regions on XrrA. These regions included sections of XrrA that are predicted to be at least partially single-stranded (Supplementary Figure 2). The predicted secondary structure of XrrA suggests formation of three hairpin loops: the 3′ terminator described earlier, a major hairpin encompassing most of the XrrA sequence, and a minor hairpin directly preceding the predicted terminator (Supplementary Figure 2). Seed region #1, which encompasses nucleotides +17 and +34 in relation to the transcriptional start site, as well as seed region #2, which includes nucleotides +72 to +96, are found along the major hairpin of XrrA. Both regions include loops of single-stranded nucleotides that would be available to mediate initial interaction with mRNA targets. One transcript is predicted to be targeted by seed region #1, while three transcripts had predicted complementarity to seed region #2 (Table 4). The third seed region was located between nucleotides +160 and +182 and is found within the 3′ hairpin terminator of XrrA. Three transcripts are predicted to base-pair with XrrA at this region, including the highly-regulated *inhA1* (Table 4).

XrrB showed limited complementarity to sRNA targets. There was no complementarity found to GBAA_0594, the only transcript differentially regulated in the Δ*xrrB* mutant by a ≥4.0 fold-change. Instead, we found that XrrB is predicted to interact with GBAA_2549, at the 5′ UTR of the transcript (Table 4). Interestingly, expression of this transcript increases 10-fold in the Δ*xrrA*Δ*xrrB* mutant, and only 3.5- and 2.0-fold in the Δ*xrrA* and Δ*xrrB* mutants, respectively, suggesting a synergistic effect of the sRNAs by an unknown mechanism. Given that XrrB is predicted to base-pair with GBAA_2549 at the 5′ UTR, repression of this transcript in the Δ*xrrA*Δ*xrrB* mutant may be, at least in part, given by decreased stability of the transcript upon interaction with XrrB.

The predicted structure of XrrB was similar to that of XrrA. This sRNA is also predicted to form three hairpins along its sequence (Supplementary Figure 3). These included the 3′ hairpin terminator, an initial hairpin located at the 5′ end of XrrB, and a major hairpin between the 5′ and 3′ hairpins, occluding a long stretch of XrrB sequence in a double-stranded structure. Interestingly, complementarity to GBAA_2549 was found at the 3′ terminator of XrrB, further illustrating the molecular similarities between XrrA and XrrB.

To further validate the base-pairing predictions, we used IntaRNA as an additional RNA-RNA interaction program (Busch et al., 2008). IntaRNA confirmed five of the seven predicted interactions between XrrA and target mRNA sequences obtained from TargetRNA2, including at least one base-pairing interaction per predicted seed region. Additionally, IntaRNA predicted a base-pairing interaction between XrrB and the GBAA_2549 mRNA, which was also predicted by TargetRNA2.

### Role of sRNAs in *B. anthracis* Virulence in a Mouse Model for Systemic Anthrax

**FIGURE 8:**
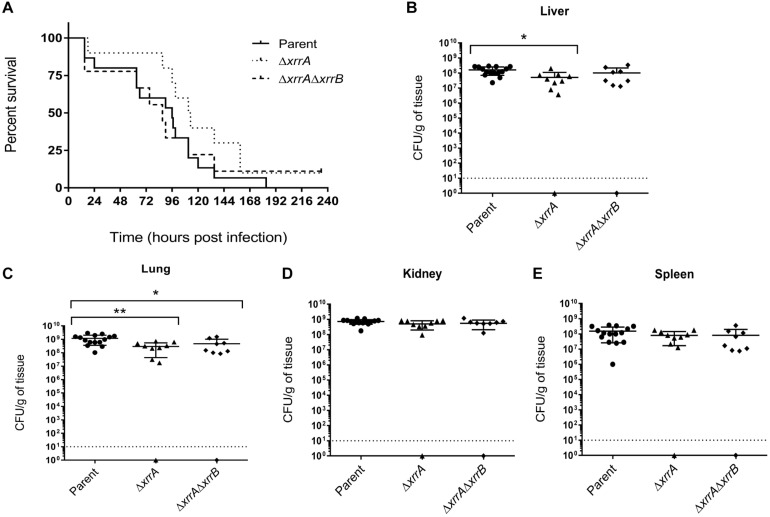
Effect of sRNA deletions on virulence of *B. anthracis* in a murine model for systemic anthrax. Seven-week-old female A/J mice were infected intravenously via the tail-vein with ∼10^5^ CFU of the ANR-1 parent strain (*n* = 15), the Δ*xrrA* mutant (*n* = 10), or the Δ*xrrA*Δ*xrrB* mutant (*n* = 9). Mice were monitored for a period of 11 days. Moribund mice were sacrificed, time of death recorded, and liver, lung, kidney, and spleen were collected for CFU determinations to measure organ infection burden. **(A)** Survival analysis using the Kaplan–Meier estimate was used to determine significance between survival of ANR-1-infected and sRNA-null-infected mice. One Δ*xrrA*-infected mouse, and one Δ*xrrA*Δ*xrrB*-infected mouse survived the 11-day period. All other mice succumbed to the infection. CFU/g of tissue of **(B)** liver, **(C)** lung, **(D)** kidney, and **(E)** spleen per infecting strain was calculated. The limit of detection is shown as a dashed line at 10^1^ CFU. The surviving Δ*xrrA*-infected and Δ*xrrA*Δ*xrrB*-infected mice showed no detectable CFU in the collected organs and are shown below the limit of detection at 10^0^. Analysis of variance (ANOVA) followed by Tukey’s multiple comparisons analysis was used to determine significance between the CFU/g of tissue of each organ for ANR-1-infected, Δ*xrrA*-infected, and Δ*xrrA*Δ*xrrB*-infected mice. ^∗^ indicates < 0.05; ^∗∗^ indicates < 0.01.

In a murine model for systemic anthrax in which complement-deficient A/J mice are infected intravenously with pXO1^+^ pXO2^–^ strains of *B. anthracis*, deletion of *atxA* leads to complete attenuation of virulence (Dai et al., 1995; Dale et al., 2012). Considering that the sRNAs are positively controlled by AtxA, we sought to determine if sRNA expression influences virulence in this model (Figure 8). We chose to infect mice with the pXO1^+^ pXO2^–^ ANR-1 strain and isogenic Δ*xrrA*, and Δ*xrrA*Δ*xrrB* mutants. XrrA regulates more targets than XrrB, including many genes exhibiting AtxA-regulated expression. The Δ*xrrA*Δ*xrrB* mutant displayed additional effects on gene expression, including highly increased expression of a putative transcriptional regulator. Mice were infected with vegetative cells of the parent and mutant strains and monitored for up to 11 days. Time to death was recorded, and organ tissues were collected for assessment of infection burden. As shown in Figure 8A, there was no statistical difference in the time to death for the parent- and mutant-infected mice. All parent-infected mice and most mutant-infected mice succumbed to infection within the 11-day period. One Δ*xrrA*-infected and one Δ*xrrA*Δ*xrrB*-infected mouse survived. These surviving mice presented no symptoms and were sacrificed at the end of the experiment.

We determined the bacterial burden in organ tissues extracted from all mice that succumbed to infection and in tissues from mice that were sacrificed on day 11. For parent-infected mice, we recovered an average of approximately 10^8^ CFU per gram of tissue from the spleen and liver, and approximately 10^9^ CFU per gram of tissue from the lungs and kidney. The surviving mutant-infected mice had no detectable CFU in their organ tissue, but all other mice that succumbed to the infection had recoverable levels of CFU. There was no difference in bacterial burden in the kidneys (Figure 8D) and spleens (Figure 8E) of parent-infected and mutant-infected mice. However, there was a statistically significant decrease in bacterial burden of the liver (Figure 8B) and lung (Figure 8C) for the Δ*xrrA* mutant compared to the parent strain. Mice that succumbed to infection with the Δ*xrrA*Δ*xrrB* mutant also showed a statistically significant decrease in the number of bacteria in the lung (Figure 8C), however, the number of bacteria in the liver (Figure 8B) was comparable to that observed for mice infected with the parent strain. The data suggest that de-regulation of XrrA targets leads to a small decrease in colonization of certain host tissues at time of death. Finally, we note that although previous reports have shown that deletion of *atxA* has minimal effects on *in vitro* growth (Dai et al., 1995; Dale et al., 2012), we observed no significant differences in growth rate between the ANR-1 parent strain and the sRNA-null mutants when cultured in CA-CO_2_ (Supplementary Figure 6).

## Discussion

In this work, we provide experimental evidence for sRNA-mediated regulation in the mammalian pathogen *B. anthracis*. The *xrrA* locus is within the 44.8-kb pathogenicity island on the virulence plasmid pXO1. The *xrrB* locus is also on pXO1, just outside of the pathogenicity island. In a previous study reporting expression of these sRNAs, it was proposed that the boundaries of pXO1 pathogenicity island be expanded to include *xrrB* (McKenzie et al., 2014). Sequence analysis using the NCBI Basic Local Alignment Search Tool (BLAST), indicates a high degree of conservation of the sRNA loci across *B. anthracis* strains and in closely-related species carrying pXO1-like plasmids. The *B. anthracis* Ames strain XrrA and XrrB sequences are 99–100% identical to the corresponding sRNAs of all other pXO1^+^ *B. anthracis* strains deposited in the NCBI genome database. The sRNA sequences are also highly conserved (99–100% identical) in the pXO1-like plasmids pBCXO1 and pCI-XO1, found in *B. cereus* strain G9241 and *B. cereus biovar anthracis* strain CI, respectively. Sequences surrounding the sRNA loci in these plasmids, as well as the anthrax toxin and *atxA* genes, are also 99–100% identical to the corresponding sequences on pXO1. These *B. cereus* strains carrying pXO1-like plasmids cause anthrax-like disease (Hoffmaster et al., 2006; Brézillon et al., 2015). The presence of both sRNAs either in proximity or within the classical pXO1 pathogenicity island, and the high degree of conservation of these loci on pXO1 plasmids, suggest co-acquisition of sRNA loci with virulence genes.

Most reports of sRNA expression and function have concerned chromosome-encoded sRNAs. Of the relatively few plasmid-encoded sRNAs that have been reported, most belong to a specific family of regulatory small RNAs called antisense RNAs. These antisense RNAs are encoded in the DNA strand directly opposite to their cognate mRNA target, resulting in long stretches of perfect complementarity between the sRNA and the target (Georg et al., 2009; Güell et al., 2009). Antisense RNAs often regulate plasmid carriage by participating in toxin-antitoxin systems and control of plasmid conjugation. Typically antisense RNAs can only base-pair to one mRNA target (van Biesen et al., 1993; Fozo et al., 2010; Arthur et al., 2011). On the other hand, chromosome-encoded sRNAs typically belong to the *trans*-encoded RNA family. These sRNAs are transcribed from a location in the bacterial genome that is distant from their cognate mRNA target and mediate base-pairing via a short seed region (Gottesman and Storz, 2011). Thus, *trans*-encoded sRNAs can typically base-pair to multiple mRNA targets using the same seed region. Despite the plasmid loci of XrrA and XrrB, together these regulatory RNAs control multiple target genes on the chromosome. Expression of a few pXO1-encoded transcripts is altered in sRNA-null mutants, but the sequences of these targets do not indicate antisense function of the sRNAs. Our data indicate that XrrA and XrrB are rare examples of *trans*-encoded sRNAs located on a plasmid. These observations place these RNAs, particularly XrrA, at the center of crosstalk between two of the *B. anthracis* genetic elements. We know of only one other report of a virulence plasmid-encoded sRNA regulating chromosomal genes; QfsR, encoded by the tumor inducing (Ti) plasmid of *Agrobacterium fabrum*, controls polycistronic mRNAs of chromosome genes involved in flagella synthesis and Ti plasmid genes associated with conjugative transfer (Diel et al., 2019).

In addition to the locations of the sRNAs on pXO1, PCVR control of XrrA and XrrB suggests a relationship between the regulatory RNAs and virulence. Our northern blotting data, as well as previous RNA-seq studies (McKenzie et al., 2014; Raynor et al., 2018), indicate that AtxA is the major regulator of XrrA and XrrB expression. While our previous report indicated that artificially expressing AcpA in the Δ*atxA*Δ*acpA*Δ*acpB* strain allowed XrrB expression (Raynor et al., 2018), here we determined that deletion of *acpA* does not affect XrrB expression, indicating a dominant role for AtxA in transcriptional control of XrrB. The crystal structure of AtxA contains two helix-turn-helix domains proximal to the amino-terminus, indicative of DNA-binding activity (Hammerstrom et al., 2015; McCall et al., 2019), and a recent report demonstrated AtxA binding to DNA sequences 5′ of *pagA*, an anthrax toxin gene (McCall et al., 2019). Nevertheless, consensus sequences for AtxA-binding to promoter regions of target genes have not been identified. We utilized the MEME suite web server (Bailey et al., 2009) to analyze sequences in the promoter regions of XrrA and XrrB and to compare them to sequences upstream of other AtxA-regulated genes. No consensus sequences were apparent despite our data showing that sRNA expression is dependent upon AtxA. It is possible that AtxA does not directly control *xrrA* and *xrrB* expression, as has been postulated for other genes in the AtxA regulon (Raynor et al., 2018).

Other examples of relationships between PCVRs and sRNAs have been reported. In *Streptococcus pyogenes*, the multiple gene activator Mga is a PCVR crucial for expression of virulence factors such as M-protein, streptococcal peptidases, and fibronectin-binding protein (Ribardo and McIver, 2006; Hondorp et al., 2013). Interestingly, expression of the *mga* gene itself is influenced by two sRNAs. The Mga-activating regulatory sRNA MarS was identified in a bioinformatic screen and found to influence expression of virulence factors of *S. pyogenes* (Pappesch et al., 2017). Deletion of *marS* leads to reduced levels of *mga* transcript. A second sRNA, RivX, also positively influences expression of Mga (Roberts and Scott, 2007). RivX is co-transcribed with a second PCVR of *S. pyogenes*, the regulator RivR. Despite co-regulation, RivX and RivR function in independent regulatory pathways and do not influence expression of each other (Roberts and Scott, 2007). In contrast to sRNA control of *mga* transcription, *atxA* transcript levels are unaffected by XrrA and XrrB. Rather, AtxA positively controls sRNA expression. To our knowledge, our study is the first report of PCVR-mediated regulation of sRNAs.

Our half-life determination experiments suggest that XrrA and XrrB are highly stable. Many sRNAs are stabilized by RNA chaperones such as Hfq. While Hfq plays a major role in sRNA function in Gram-negative bacteria, contributions of Hfq to sRNA-mediated regulation vary between species in Gram-positive bacteria. We found that XrrA and XrrB stability is unaffected by deletion of any of the *B. anthracis hfq* genes. Interestingly, evidence for RNA chaperones other than Hfq has emerged in recent years. The highly conserved bacterial ProQ protein, first studied in *E. coli*, stabilizes sRNAs and facilitates base-pairing to mRNA targets, similarly to Hfq (Olejniczak and Storz, 2017). The RNA-binding protein CsrA, which regulates carbon catabolism by influencing mRNA translation and decay, facilitates interactions between sRNAs and their mRNA targets in *B. subtilis* (Müller et al., 2019). Another highly conserved protein, YbeY, influences expression of sRNAs in *E. coli* and accumulation of sRNAs in the plant symbiont *Sinorhizobium meliloti* (Pandey et al., 2011, 2014). It is possible that the sRNAs of *B. anthracis* are stabilized by proteins other than Hfq. The molecular features of XrrA and XrrB may also provide stability. In Gram-positive bacteria, the endonucleolytic enzyme RNase Y mediates the first rate-limiting internal cleavage of transcripts, followed by processive degradation mediated by exonucleases (Mohanty and Kushner, 2016). Additionally, 5′ to 3′ exonucleolytic decay may be initiated by the 5′ exonuclease complex RNase J1/J2 (Even et al., 2005; Bechhofer, 2011). The catalytic activity of RNases Y and J1/J2 is enhanced by a 5′ monophosphate group at the 5′ of transcripts (Even et al., 2005; Li de la Sierra-Gallay et al., 2008; Shahbabian et al., 2009; Bandyra et al., 2012; De Lay and Gottesman, 2012). Our data indicate that both XrrA and XrrB are primary transcripts, each with a 5′ triphosphate group. Thus, the sRNAs are predicted to be protected from RNase Y-mediated endonucleolytic cleavage and RNase J1/J2-mediated 5′ exonucleolytic decay. Moreover, the predicted hairpin terminators at the sRNA 3′ ends would confer protection against 3′ exonucleolytic decay. Additionally, most of the sRNA sequences are predicted to be occluded by extensive secondary structure, which would further protect the sRNAs from RNase Y, which requires single-stranded structure for cleavage. Initiation of XrrA and XrrB decay in *B. anthracis* would likely be dependent on endonucleases that specifically target double-stranded regions on RNA, such as RNase III, which has a minor role in bulk RNA turnover compared to RNase Y and RNase J1/J2 (Durand et al., 2012). While RNA decay has not been studied directly in *B. anthracis*, we infer from RNA decay mechanisms of other species that XrrA and XrrB are highly stable due in part to their 5′ and 3′ end characteristics and their extensive secondary structure.

Our RNA-seq data show that multiple transcripts are affected by deletion of *xrrA* alone. Given that seven of those transcripts are predicted to directly interact with XrrA, it is likely that this sRNA functions by base-pairing. We found three predicted seed regions on XrrA, predicted to contain short single-stranded regions. Initial base-pairing between these regions and the mRNA targets could result in rearrangement of the XrrA secondary structure, revealing further single-stranded stretches to complete base-pairing along the entire seed region. Furthermore, levels of some transcripts are altered in the sRNA double-deletion, but are unaffected in single sRNA-null mutants, suggesting some functional overlap between XrrA and XrrB. Particularly, expression of the GBAA_3479 transcript predicted to encode a member of the ArsR family of transcriptional regulators, was highly increased in the double-null only, suggesting that both sRNAs are required for regulation of this target. We found no sequence complementary between the sRNAs and GBAA_3479, so direct base-pairing to GBAA_3479 by both sRNAs seems unlikely. The additional 10 transcripts that were altered only in the double mutant may be responsive to the GBAA_3479 gene product.

It is possible that the sRNAs may also function as protein-interacting sRNAs and compete for interaction with the same regulatory protein(s). There are several examples of multiple sRNAs titrating the same proteins. For example, the multiple sRNAs of the *P. aeruginosa* Rsm system titrate the regulatory proteins RsmA and RsmF (Vakulskas et al., 2015; Miller et al., 2016; Janssen et al., 2018). Interactions between the sRNAs and regulatory proteins of *B. anthracis* could account for the apparent overlapping functions of XrrA and XrrB. Future experiments will test for direct base-paring between the sRNAs and target gene sequences, as well as for sRNA-protein interactions.

Gene ontology analysis suggests at least two groups of XrrA-regulated genes that are of particular interest for a pathogen that survives in multiple host tissues. First, XrrA regulates expression of several proteases, including InhA1, CalY, and the putative aminopeptidase GBAA_5606. Second, XrrA also regulates expression of predicted amino acid transporters, including the predicted BCAA transporter BrnQ3, and the predicted oligopeptide transporters GBAA_0656, GBAA_0658, and GBAA_0852. Co-regulation of these targets by XrrA suggests a link between expression of proteases during an infection and acquisition of amino acids for growth and survival, in agreement with a previously proposed model (Terwilliger et al., 2015). In collaboration with Terwilliger et al. (2015) we reported that *B. anthracis* requires valine for growth in a synthetic medium designed to mimic human serum (BSM medium). In our experiments, synthesis of the InhA1 protease allowed *B. anthracis* to grow in BSM lacking valine, likely due to acquisition of valine from proteolysis of serum proteins (Terwilliger et al., 2015). If indeed valine and other amino acids can be obtained from the breakdown of host proteins by InhA1, the resulting oligopeptides would enter *B. anthracis* cells via membrane-bound oligopeptide importers or permeases. XrrA-mediated control of both InhA1 and predicted BCAA and oligopeptide transporters, as shown in work reported here, further supports the model for acquisition of BCAAs and other nutrients from proteolyzed host proteins.

Although we did not observe sRNA-associated growth rate, colony morphology, or sporulation when parent and mutant strains were cultured in various media (data not shown), we predicted that altered expression of the large and diverse XrrA regulon would affect *B. anthracis* pathogenesis in an animal model for anthrax. Although our animal experiments did not reveal changes in time to death, the *xrrA*-null mutant showed reduced numbers of bacteria in the murine liver and lungs at time of death. A relationship between the model for BCAA acquisition and tissue-specific differences in colonization is worthy of speculation. Quantification of BCAA concentrations in the blood and lungs of humans and pigs suggest varying levels of BCAA availability, such that BCAA levels are higher in blood than in lungs (Subashchandrabose et al., 2009; Kaiser and Heinrichs, 2018). Additionally, activity of the first two enzymes involved in BCAA catabolism is highest in human liver and skeletal muscle tissues, leading to high utilization and low accumulation of BCAAs (Holeček, 2018). Thus, liver and lung niches appear to be BCAA-poor. Given that BCAA availability varies across host niches within the mammalian host, tight regulation of BCAA acquisition could be a determinant of pathogen success. *B. anthracis*, which replicates to high titers in the blood and can colonize virtually all host tissues, may need to fine-tune degradation of host proteins for uptake of host-derived BCAAs according to nutrient availability in different niches. While this model requires further exploration, our data indicate that XrrA co-regulates amino acid transport, amino acid biosynthesis, and protease expression, and that dysregulation of these XrrA targets leads to decreased bacterial abundance in BCAA-poor host niches.

XrrA and XrrB are the first reported sRNAs of *B. anthracis* and are rare examples of plasmid-borne *trans*-acting sRNAs. Also, to our knowledge XrrA and XrrB are the only sRNAs reported to be controlled by a PCVR. Together, these highly expressed stable sRNAs play significant roles in regulatory crosstalk between the pXO1 virulence plasmid and the *B. anthracis* chromosome. The large XrrA regulon controls expression of genes associated with *B. anthracis* virulence and metabolic genes that may be important for bacterial-host interaction. Future work will focus on the molecular basis for sRNA function, including investigations of potential RNA and/or protein interacting partners of XrrA and XrrB, and studies of the function of specific sRNA-controlled genes in *B. anthracis* physiology and virulence.

## Data Availability Statement

The datasets presented in this study can be found in online repositories. The names of the repository/repositories and accession number(s) can be found in the article/Supplementary Material.

## Ethics Statement

The animal study was reviewed and approved by Institutional Biosafety and Animal Welfare Committees of the University of Texas Health Science Center – Houston.

## Author Contributions

IC and TK contributed to the conception and design of the study. IC, SD, AV, and TK contributed to data acquisition, analysis, and interpretation of the data. IC and TK wrote the article. All authors contributed to the article and approved the submitted version.

## Conflict of Interest

The authors declare that the research was conducted in the absence of any commercial or financial relationships that could be construed as a potential conflict of interest.

## Footnotes

1 https://usegalaxy.org/

2 https://www.ncbi.nlm.nih.gov/geo/query/acc.cgi?acc=GSE152356
