# Supplementary material for: AtxA-Controlled Small RNAs of Bacillus anthracis Virulence Plasmid pXO1 Regulate Gene Expression in trans
Source: Front Microbiol. 2021 Jan 15;11:610036. doi: 10.3389/fmicb.2020.610036 (PMC7843513; doi:10.3389/fmicb.2020.610036)
Supplement: Supplementary file 1 [file Image_1.pdf]

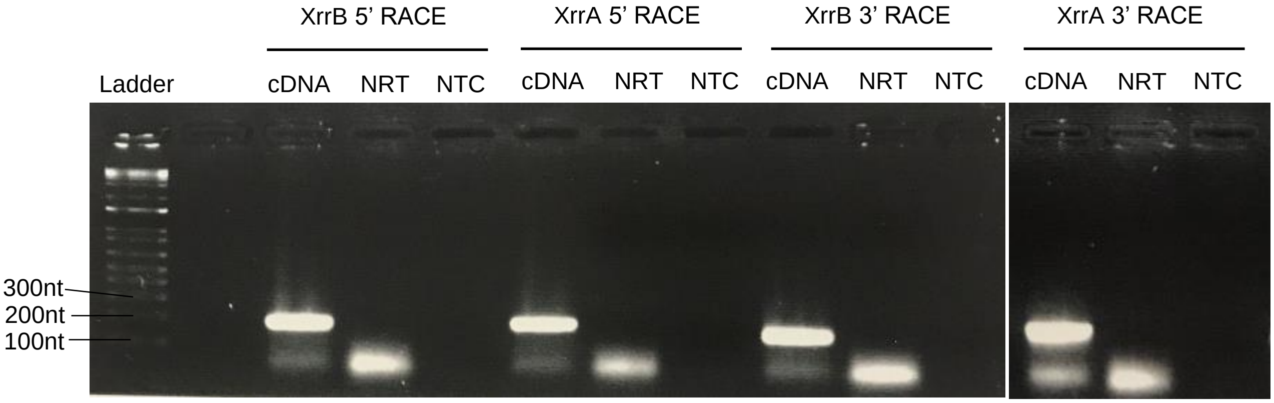

FIGURE S1 RACE analysis of sRNA 5' and 3' ends. Total RNA isolated from the ANR-1 parent strain was treated with DNase I to remove potential genomic DNA contamination. For 5' RACE, RNA was treated with RNA 5' Polyphosphatase (5'PP) and then ligated to the 5' RACE Adapter by incubation with T4 RNA Ligase I. cDNA synthesis of the ligated RNA was performed and followed by PCR analysis using 5' RACE Adapter-specific and sRNA-specific primers. For 3' RACE, DNase-treated RNA was incubated with Calf Intestinal Alkaline Phosphatase (CIP) and ligated to the 3' RACE Adapter using T4 RNA Ligase I. After cDNA synthesis, PCR analysis of the ligated RNA was performed using 3' RACE Adapter-specific and sRNA-specific primers. No reverse transcriptase (NRT) RNA and no template (NTC) controls were included for each primer set. PCR products were visualized on a 1.5% agarose gel. Products were excised from the gel and subjected to Sanger sequencing for identification of 5' and 3' ends at the ligated junction between the RACE Adapters and the sRNAs.
