# Supplementary material for: AtxA-Controlled Small RNAs of Bacillus anthracis Virulence Plasmid pXO1 Regulate Gene Expression in trans
Source: Front Microbiol. 2021 Jan 15;11:610036. doi: 10.3389/fmicb.2020.610036 (PMC7843513; doi:10.3389/fmicb.2020.610036)
Supplement: Supplementary file 2 [file Image_2.pdf]

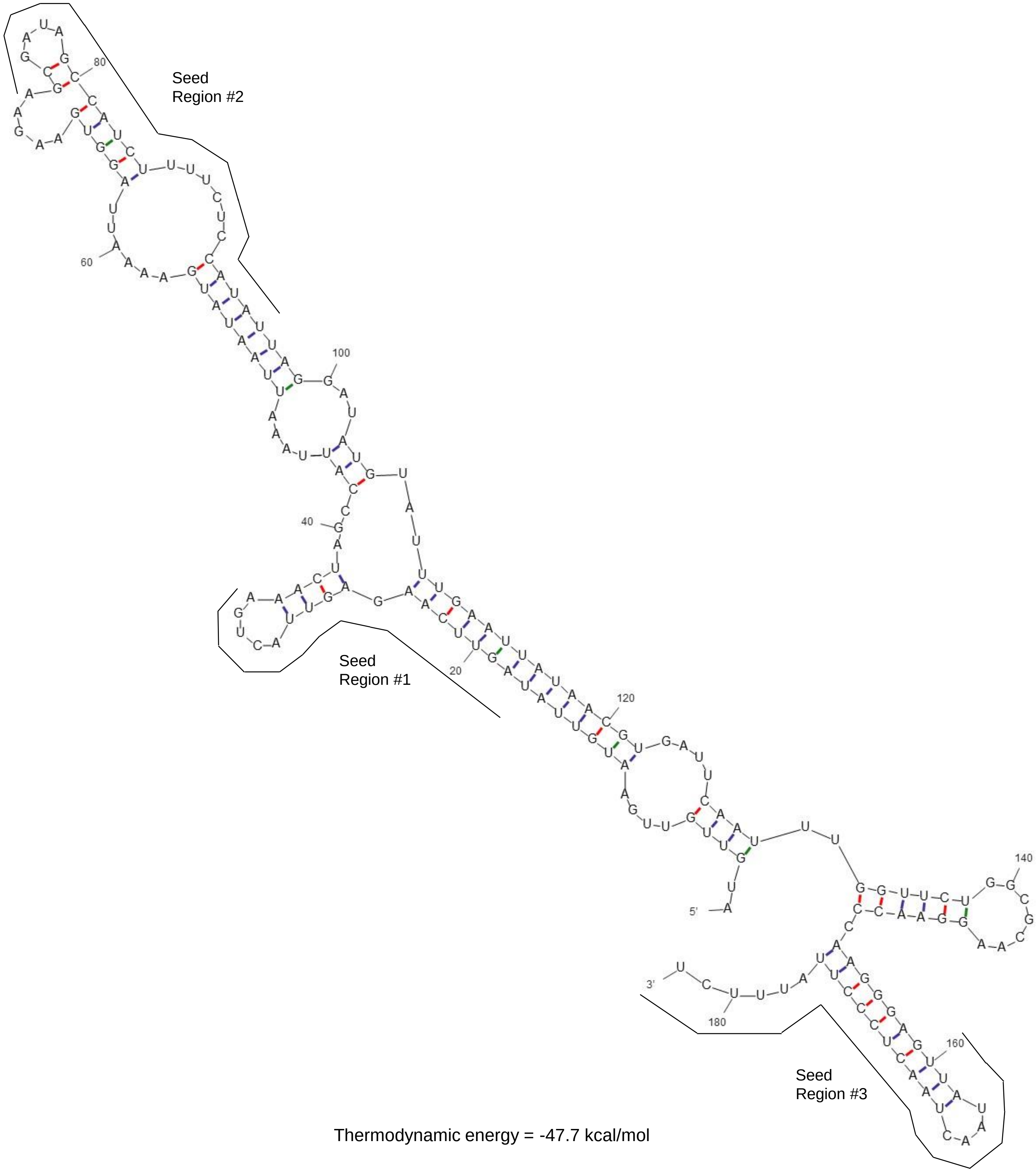

FIGURE S2 Predicted XrrA structure and thermodynamic energy (kcal/mol) of folding, as calculated by the mfold webserver with default parameters. The predicted seed regions of XrrA, uncovered from the TargetRNA2 analysis, are labeled.
