# Supplementary material for: AtxA-Controlled Small RNAs of Bacillus anthracis Virulence Plasmid pXO1 Regulate Gene Expression in trans
Source: Front Microbiol. 2021 Jan 15;11:610036. doi: 10.3389/fmicb.2020.610036 (PMC7843513; doi:10.3389/fmicb.2020.610036)
Supplement: Supplementary file 4 [file Image_4.pdf]

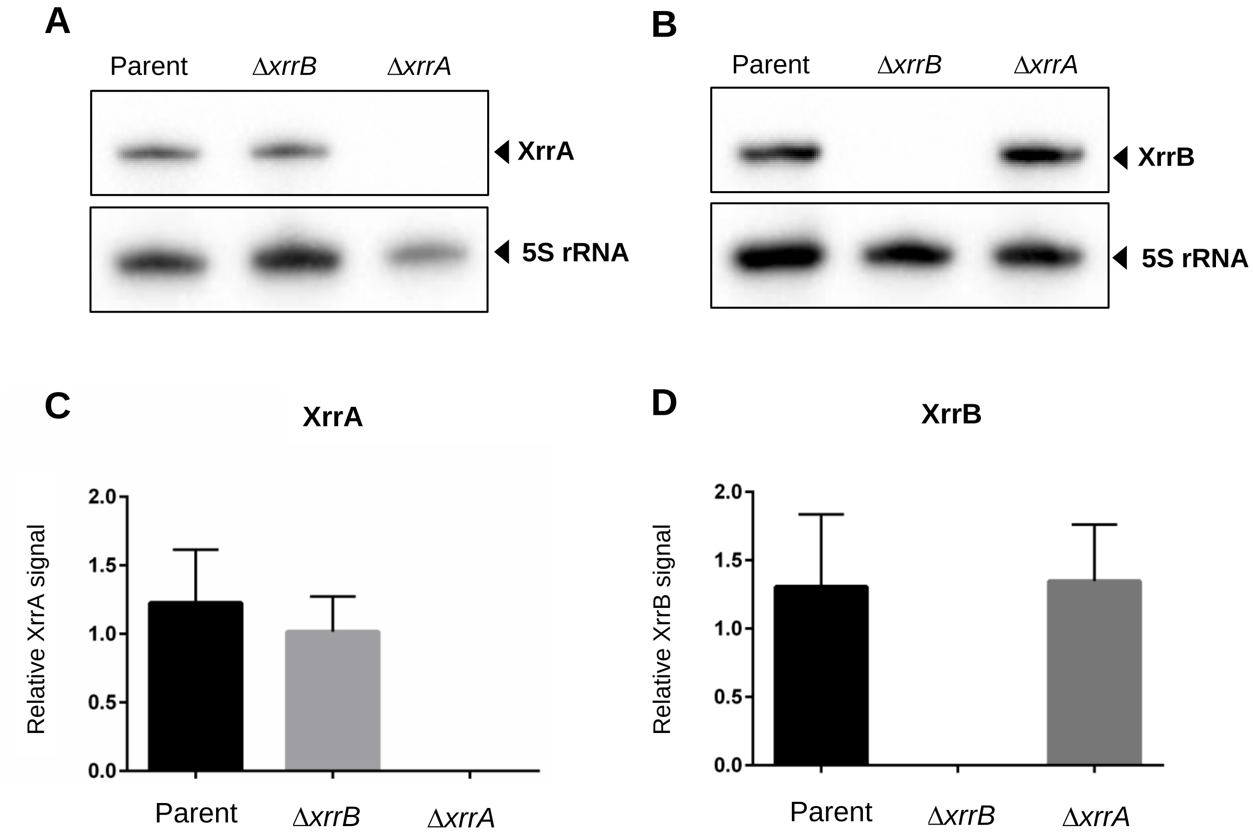

FIGURE S4 Effect of XrrA and XrrB deletions on expression of each sRNA. The ANR-1 parent strain, the Δ*xrrA* mutant, and the Δ*xrrB* mutant were grown in CA-CO_2 until early stationary phase (OD_{600} = 1.0 – 1.5). RNA was extracted and subjected to northern blotting. **(A)** XrrA and **(B)** XrrB expression in the parent ANR-1 strain and the sRNA-null mutants is shown, with 5S rRNA as a load control. Northern blots shown are representative images from three biological replicates. Quantification of **(C)** XrrA and **(D)** XrrB expression was calculated from averaged data as a fraction of sRNA expression normalized to 5S rRNA in the parent strain. The standard deviation in sRNA expression across the strains is shown. An Analysis of Variance (ANOVA) followed by Tukey's multiple comparison analysis was utilized to determine whether sRNA expression is significantly different between the parent and other sRNA-null strain.
