# Supplementary material for: AtxA-Controlled Small RNAs of Bacillus anthracis Virulence Plasmid pXO1 Regulate Gene Expression in trans
Source: Front Microbiol. 2021 Jan 15;11:610036. doi: 10.3389/fmicb.2020.610036 (PMC7843513; doi:10.3389/fmicb.2020.610036)
Supplement: Supplementary file 5 [file Image_5.pdf]

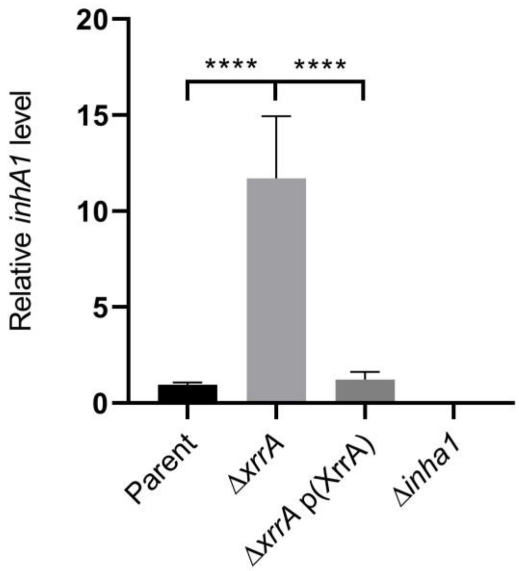

FIGURE S5 Validation of XrrA-mediated *inhA1* regulation. The Ames-derived parent strain, the xrrA-null, the XrrA complementation strain, and the inhA1-null were grown in CA-CO_2 until early stationary phase (OD_{600} = 1.0 – 1.5) and RNA was extracted for cDNA synthesis. CT values for *inhA1* and the reference gene *gyrB* were obtained using SYBR-Green qPCR and gene-specific primers. Average relative *inhA1* level from three biological replicates is shown. Error bars represent standard deviation. Analysis of variance (ANOVA) followed by Tukey's multiple comparison test was used to determine significance. **** indicates < 0.0001.
