# Supplementary material for: AtxA-Controlled Small RNAs of Bacillus anthracis Virulence Plasmid pXO1 Regulate Gene Expression in trans
Source: Front Microbiol. 2021 Jan 15;11:610036. doi: 10.3389/fmicb.2020.610036 (PMC7843513; doi:10.3389/fmicb.2020.610036)
Supplement: Supplementary file 6 [file Image_6.pdf]

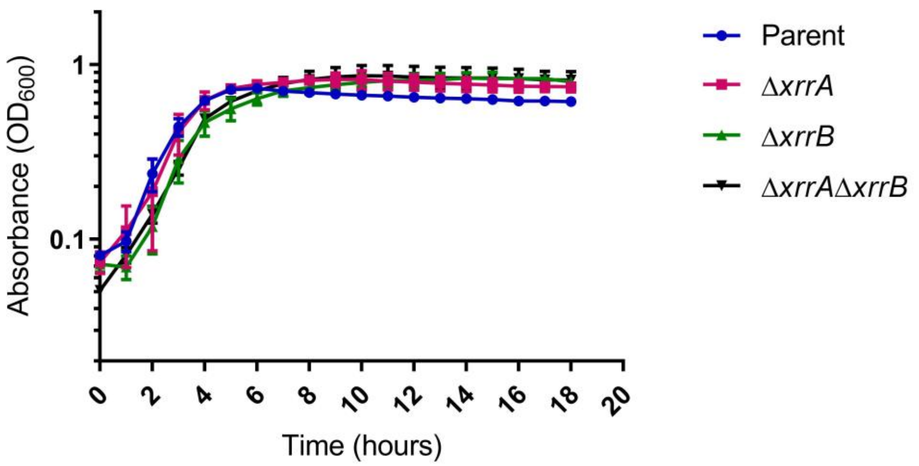

FIGURE S6 Effect of sRNA deletions on growth of *B. anthracis* in CA-CO_2. The ANR-1 parent strain, the Δ*xrrA* mutant, the Δ*xrrB* mutant, and the Δ*xrrA*Δ*xrrB* mutant were grown in 1 ml of CA medium, at a starting OD_{600} of 0.08 in a 26-well plate. Cells were grown in a plate reader at 37°C with continuous orbital shaking at 355 c.p.m., in the presence of 5% CO_2. Absorbance measurements (OD_{600}) were taken every hour for 18 hours. The average absorbance at each time-point for three biological replicates and the standard deviation for each time-point are shown.
