# Supplementary material for: AtxA-Controlled Small RNAs of Bacillus anthracis Virulence Plasmid pXO1 Regulate Gene Expression in trans
Source: Front Microbiol. 2021 Jan 15;11:610036. doi: 10.3389/fmicb.2020.610036 (PMC7843513; doi:10.3389/fmicb.2020.610036)
Supplement: Supplementary file 7 [file Table_1.docx]

| **TABLE S1** Primers and oligos used in this study. | | | | | |
| --- | --- | --- | --- | --- | --- |
| **Name** | | **Sequence^a^** | | **Function** | |
| **Primers** | |  | |  | |
| NB094 | | AAACTGCAGCAATAAATCCGCCTAAATTAAAATTAAGATCTATAATTG | | *xrrA* deletion construct, 1kb downstream of *xrrA* locus | |
| NB095 | | TATAACTCCCCAACATAGCTAAAATATACATCAATTTATAAAGGGG | | Overlap extension of *xrrA* deletion construct | |
| NB096 | | GCTATGTTGGGGAGTTATAACTAACTCCCTTATTTCT | | Overlap extension of *xrrA* deletion construct | |
| NB097 | | AAAGTCGACCATTTCATATATTCACATTATAATATCAACATCATGC | | *xrrA* deletion construct, 1kb upstream of *xrrA* locus | |
| NB088 | | AAACTGCAGGAATATATCTCTAACCTTATTGTTAATTTTTGATATTTCG | | *xrrB* deletion construct, 1kb downstream of *xrrB* locus | |
| NB089 | | TGTATTATTTAGGTTAATTAAAAATACCTAAAACAAATGCTGTTTTAGG | | Overlap extension of *xrrB* deletion construct | |
| NB090 | | TTTTTAATTAACCTAAATAATACAATCAATATGATGTAGAGCGG | | Overlap extension of *xrrB* deletion construct | |
| NB091 | | AAAGTCGACCAATGATTTGATGGAACGTCCAGA | | *xrrB* deletion construct, 1kb upstream of *xrrB* locus | |
| IC23 | | AAAGTCGACGAAGTGCGGAATAAGAAACTGAC | | *hfq1* deletion construct, 1kb downstream of *hfq1* locus | |
| IC24 | | CGAGTGTCTTACATCTCTCTCCTTTTTGTTTAATATC | | Overlap extension of *hfq1* deletion construct | |
| IC25 | | GGAGAGAGATGTAAGACACTCGTGCTGAGTGT | | Overlap extension of *hfq1* deletion construct | |
| IC26 | | AAACTGCAGCTTCTAAAGAAAGTAGGCCGTG | | *hfq1* deletion construct, 1kb upstream of *hfq1* locus | |
| IC49 | | AAAGTCGACTAATTCTGCTTCATTCATTCAAG | | *hfq2* deletion construct, 1kb downstream of *hfq2* locus | |
| IC50 | | GGAGGATTCGACGAGTAGTAATGAATTATTGTACATAAC | | Overlap extension of *hfq2* deletion construct | |
| IC51 | | TCATTACTACTCGTCGAATCCTCCTCTTTTTCT | | Overlap extension of *hfq2* deletion construct | |
| IC52 | | AAACTGCAGATGTACCGTAATGAAAGAACTT | | *hfq2* deletion construct, 1kb upstream of *hfq2* locus | |
| IC01 | | AAAGTCGACCGATGAAATTGAACATGCTGAAT | | *hfq3* deletion construct, 1kb downstream of *hfq3* locus | |
| IC02 | | TTTTAGATGGTCATGAATAGTTTCTTCCTCTC | | Overlap extension of *hfq3* deletion construct | |
| IC03 | | AACTATTCATGACCATCTAAAAGTCTAATTTG | | Overlap extension of *hfq3* deletion construct | |
| IC04 | | AAACTGCAGGGATGAAGCTACCTCCATGTG | | *hfq3* deletion construct, 1kb upstream of *hfq3* locus | |
| IC91 | | AAAGCATGCAGAAATAAGGGAGTTAGTTATAACTCCCTTGGG | | *xrrA* expression construct for ligation to pUTE657 | |
| IC92 | | AAAGTCGACATGTTGTTGAATGTTATAGTTCAAGAG | | *xrrA* expression construct for ligation to pUTE657 | |
| IC108 | | GTTCAAGAGTTACTGAAACTAGCC | | *xrrA* DNA probe template | |
| **TABLE S1** (Continued) | | | | | |
| **Name** | | **Sequence** | | **Function** | |
| IC109 | | CCATACCCATACATTTAATTTTAG | | *xrrA* DNA probe template | |
| IC106 | | GGGGAAACGTAGGGATTTAAAC | | *xrrB* DNA probe template | |
| IC107 | | CAACGCTTACAAATAATGTAAGTG | | *xrrB* DNA probe template | |
| IC31 | ATTTGCCTGGCAACGTCCTAC | | 5S rRNA DNA probe template | |  |
| IC32 | GAATACAGTCTGGTAATGATGGC | | 5S rRNA DNA probe template | |  |
| IC200 | GCTCTTATGAAGTTAGCGGCGGAC | | 16S rRNA DNA probe template | |  |
| IC201 | CCACTTTCCTCTTCTGCACTCAAG | | 16S rRNA DNA probe template | |  |
| IC202 | CCTGGAGGAAGAGAAAGCAAATGC | | 23S rRNA DNA probe template | |  |
| IC203 | CCCTGGACATGGGTAGATCACC | | 23S rRNA DNA probe template | |  |
| IC191 | GAGTACGTAAGTGCTCGTTTAG | | *rpsO* DNA probe template | |  |
| IC192 | CTTTAACACAAGAGCGTAAAAATG | | *rpsO* DNA probe template | |  |
| IC46 | GCGCCAGAACCAAATTGAATCACG | | *xrrA*-specific 5’ RACE primer | |  |
| IC53 | CAAATACATATCCTAATATGGAG | | *xrrA*-specific 5’ RACE primer | |  |
| IC47 | GTTCAAGAGTTACTGAAACTAGCCA | | *xrrA*-specific 3’ RACE primer | |  |
| IC48 | GAAATTAGGTGAAGAAGCGATAGCC | | *xrrA*-specific 3’ RACE primer | |  |
| IC68 | CAAGAGTTACTGAAACTAGCC | | *xrrA*-specific 3’ RACE primer | |  |
| IC54 | GTGATGCATGTTTAATAATTC | | *xrrB*-specific 5’ RACE primer | |  |
| IC55 | CATTAGACTACATACAGCTATCG | | *xrrB*-specific 5’ RACE primer | |  |
| IC69 | GTCCAGTATCTATTCTATTCAC | | *xrrB*-specific 3’ RACE primer | |  |
| IC70 | CAATTAGGCAATAAATACCATTCCC | | *xrrB*-specific 3’ RACE primer | |  |
| IC105 | GTGACAAGAATCGCCGGCGTACGAG | | 5’ RACE Adapter-specific primer | |  |
| IC104 | GAGCATGCGGCCGCTAAGAAC | | 3’ RACE Adapter-specific primer | |  |
| TH195 | GAACGTTGCTCGAGGGTAAATG | | pUTE657-specific primer | |  |
| TH196 | GGTACGTACGATCTTTCAGCC | | pUTE657-specific primer | |  |
| IC218 | GTGAAAGATGCGAAAGCAGATAC | | *inhA1*-specific qPCR primer | |  |
| IC219 | CGCTGGAGATGTTGGTACTT | | *inhA1*-specific qPCR primer | |  |
| IC214 | AAGGTGGAACACATGAAGTAGG | | *gyrB*-specific qPCR primer | |  |
| IC215 | CGAACGTCCTCACCAGTTAAA | | *gyrB*-specific qPCR primer | |  |
| **Oligos** |  | |  | |  |
| 5’ RACE  Adapter | UUCACUGUUCUUAGCGGCCGCAUGCUC | | 5’ RACE Adapter RNA oligo | |  |
| 3’ RACE  Adapter | (p)UUCACUGUUCUUAGCGGCCGCAUGCUC(dT) | | 3’ RACE Adapter RNA oligo, contains 5’ mono-phosphate and 3’ inverted dT modifications | |  |
