# Supplementary material for: AtxA-Controlled Small RNAs of Bacillus anthracis Virulence Plasmid pXO1 Regulate Gene Expression in trans
Source: Front Microbiol. 2021 Jan 15;11:610036. doi: 10.3389/fmicb.2020.610036 (PMC7843513; doi:10.3389/fmicb.2020.610036)
Supplement: Supplementary file 8 [file Table_2.docx]

| **TABLE S2** Transcripts regulated and co-regulated by the sRNAs. | | | | **Log2(fold-change)^a^** | | |
| --- | --- | --- | --- | --- | --- | --- |
| **Genetic Element** | **Transcript locus tag** | **Gene names** | **Function** | **∆*xrrA*** | **∆*xrrB*** | **∆*xrrA***  **∆*xrrB*** |
| Chr. | GBAA_3479 |  | ArsR Transcriptional Regulator | − | − | +7.43 |
| Chr. | GBAA_1295 | *inhA1* | Immune inhibitor metalloprotease | +4.52 | +1.02 | +5.63 |
| Chr. | GBAA_1132 | *aceA* | Isocitrate lyase | +4.08 | − | +5.05 |
| Chr. | GBAA_5606 |  | Putative aminopeptidase | +3.92 | − | +4.99 |
| Chr. | GBAA_2827 |  | Putative chitin binding protein | +3.21 | +0.98 | +4.89 |
| Chr. | GBAA_1854 | *ilvA* | Threonine ammonia-lyase | +2.56 | − | +4.53 |
| Chr. | GBAA_4149 |  | Putative hydrolase | +2.62 | − | +4.44 |
| Chr. | GBAA_2633 |  | Putative cysteine deoxygenase | +2.06 | − | +4.44 |
| Chr. | GBAA_3709-GBAA_3710 | *hutG-hutI* | Formiminoglutamase- imidazolonepropionase | +3.21 | − | +4.22 |
| Chr. | GBAA_1853 | *ilvD* | Dihydroxy-acid dehydratase | +2.26 | − | +4.04 |
| Chr. | GBAA_2354 | *mmsA1* | Methylmalonic acid semialdehyde dehydrogenase | +2.81 | − | +3.95 |
| Chr. | GBAA_2348 | *mmgD* | Citrate (Si)-synthase | +2.94 | − | +3.95 |
| Chr. | GBAA_2552 |  | Putative carboxyl transferase domain protein | +2.39 | − | +3.94 |
| Chr. | GBAA_3310 |  | Hypothetical protein | +3.45 | − | +3.87 |
| Chr. | GBAA_2521-GBAA_2522 |  | Putative lipoprotein-hypothetical protein | +2.69 | − | +3.86 |
| Chr. | GBAA_1131 | *aceB* | Malate synthase A | +2.98 | − | +3.75 |
| Chr. | GBAA_3854 | *chi36* | Extracellular exochitinase | +3.61 | +1.41 | +3.72 |
| Chr. | GBAA_1968-GBAA_1969-GBAA_1970 | *hom1-thrC-thrB* | Homoserine dehydrogenase-threonine synthase-homoserine kinase | +2.75 | − | +3.72 |
| Chr. | GBAA_3609 | *dhaS* | Aldehyde dehydrogenase | +2.15 | − | +3.58 |
| Chr. | GBAA_2553 |  | Putative acetoacetyl-CoA synthase | +2.16 | − | +3.56 |
| Chr. | GBAA_4690 | *valS* | Valyl-tRNA synthetase | +3.25 | − | +3.45 |
| Chr. | GBAA_3315 |  | Putative isochorismatase family protein | +2.56 | +1.21 | +3.45 |
| Chr. | GBAA_4292-GBAA_4293 |  | Putative methionine/alanine transporter-putative sodium symporter | +2.45 | − | +3.44 |
| Chr. | GBAA_4514 | *cccA* | Cytochrome c-550 | +2.16 | − | +3.41 |
| Chr. | GBAA_0302 |  | Hypothetical protein | +1.88 | − | +3.41 |
| Chr. | GBAA_3874 |  | Hypothetical protein | +1.9 | − | +3.39 |
| Chr. | GBAA_2549 |  | Hypothetical protein | +1.8 | +1.03 | +3.37 |

| **TABLE S2** (Continued) | | | | **Log2(fold-change)^a^** | | |
| --- | --- | --- | --- | --- | --- | --- |
| **Genetic Element** | **Transcript locus tag** | **Gene names** | **Function** | **∆*xrrA*** | **∆*xrrB*** | **∆*xrrA***  **∆*xrrB*** |
| Chr. | GBAA_2353 |  | Putative 2-hydroxy-3-oxopropionate reductase | +2.43 | − | +3.37 |
| Chr. | GBAA_2551 |  | Putative Enoyl-CoA hydratase/isomerase | +1.59 | − | +3.32 |
| Chr. | GBAA_3711 | *hutU* | Urocanate hydratase | +2.42 | − | +3.3 |
| Chr. | GBAA_1546 | *qcrC* | Menaquinol-cytochrome c reductase | +2.1 | − | +3.3 |
| Chr. | GBAA_2860 |  | CocE/NonD hydrolase | +1.36 | − | +3.3 |
| Chr. | GBAA_2350 | *prpB* | Methylisocitrate lyase | +2.38 | − | +3.24 |
| Chr. | GBAA_4486 |  | Hypothetical protein | +1.84 | +0.98 | +3.21 |
| Chr. | GBAA_1852 | *ilvC2* | Ketol-acid reductoisomerase | +1.52 | − | +3.2 |
| Chr. | GBAA_1459 | *brnQ3* | Branched-chain amino acid transporter | +1.9 | − | +3.18 |
| Chr. | GBAA_1420 | *leuA* | 2-isopropylmalate synthase | +3.07 | − | +3.18 |
| Chr. | GBAA_0241-GBAA_0242 |  | Putative fumarylacetoacetate hydrolase-putative homogentisate 1,2-dioxygenase | +1.7 | − | +3.11 |
| Chr. | GBAA_0598 |  | Hypothetical protein | +1.87 | − | +3.09 |
| Chr. | GBAA_3312 | *ectB* | Diaminobutyrate-2-oxoglutarate transaminase | +2.83 | − | +3.06 |
| Chr. | GBAA_2548 |  | Putative acetyl-CoA carboxylase | +1.69 | − | +3.04 |
| Chr. | GBAA_1545 | *qcrB* | Menaquinol-cytochrome c reductase | +1.81 | − | +3 |
| Chr. | GBAA_2301-GBAA_2300 | *GBAA_2301-kamA* | Hypothetical protein-L-lysine 2,3-aminomutase | +2.13 | − | +2.9 |
| Chr. | GBAA_4342 |  | Hypothetical protein | +1.68 | − | +2.88 |
| Chr. | GBAA_0093 | *sigH* | RNA polymerase sigma-H factor | +0.81 | − | +2.88 |
| Chr. | GBAA_0977 |  | Hypothetical protein | − | − | +2.87 |
| Chr. | GBAA_2352 |  | Putative acyl-CoA dehydrogenase | +1.96 | − | +2.85 |
| Chr. | GBAA_0656 |  | Putative oligopeptide ABC transporter | +3.16 | +1.52 | +2.84 |
| Chr. | GBAA_4550 |  | Hypothetical protein | +1.93 | +1.57 | +2.81 |
| Chr. | GBAA_1419 | *ilvC1* | Ketol-acid reductoisomerase | +2.49 | − | +2.78 |
| Chr. | GBAA_4468 |  | Hypothetical protein | +2.28 | − | +2.76 |
| Chr. | GBAA_2001 |  | Putative intracellular serine protease | +1.47 | − | +2.76 |
| Chr. | GBAA_1646 |  | Hypothetical protein | − | − | +2.69 |

| **TABLE S2** (Continued) | | | | **Log2(fold-change)^a^** | | |
| --- | --- | --- | --- | --- | --- | --- |
| **Genetic Element** | **Transcript locus**  **tag** | **Gene names** | **Function** | **∆*xrrA*** | **∆*xrrB*** | **∆*xrrA***  **∆*xrrB*** |
| Chr. | GBAA_2859 |  | Hypothetical protein | − | − | +2.66 |
| Chr. | GBAA_2153 |  | Hypothetical protein | +1.4 | − | +2.66 |
| Chr. | GBAA_0543 |  | Putative penicillin-binding domain protein | +0.66 | − | +2.66 |
| Chr. | GBAA_3584 |  | Putative microbial collagenase | +2.38 | − | +2.61 |
| Chr. | GBAA_4895 |  | Hypothetical protein | +1.62 | − | +2.6 |
| Chr. | GBAA_3243 |  | Putative DNA-binding protein | − | − | +2.58 |
| Chr. | GBAA_1417-GBAA_1418 | *ilvB-ilvN* | Acetolactate synthase, large subunit-acetolactate synthase, small subunit | +1.9 | − | +2.57 |
| Chr. | GBAA_2723 |  | Pseudo carboxylesterase | +1.78 | − | +2.57 |
| Chr. | GBAA_2673 |  | Putative chitosanase | +1.7 | − | +2.56 |
| Chr. | GBAA_0318 |  | MarR transcriptional regulator | +0.7 | − | +2.54 |
| Chr. | GBAA_2349 | *mmgE* | 2-methylcitrate dehydratase | +1.9 | − | +2.53 |
| Chr. | GBAA_0852 |  | Putative ABC transporter | +1.99 | +1.29 | +2.51 |
| Chr. | GBAA_1433 |  | Hypothetical protein | +1.98 | − | +2.45 |
| Chr. | GBAA_2377 | *hup2* | DNA-binding protein HU | +1.33 | − | +2.44 |
| Chr. | GBAA_4334 | *ribH* | 6,7-dimethyl-8-ribityllumazine synthase | +1.18 | − | +2.44 |
| Chr. | GBAA_2547 |  | Putative acyl-CoA dehydrogenase | +1.40 | − | +2.43 |
| Chr. | GBAA_1091 |  | Putative long-chain-fatty-acid-CoA ligase | +1.97 | − | +2.37 |
| Chr. | GBAA_4997 |  | Putative ABC transporter | +0.88 | − | +2.37 |
| Chr. | GBAA_4759 | *etfA* | Electron transfer flavoprotein | +1.33 | − | +2.36 |
| Chr. | GBAA_5523 |  | Hypothetical protein | +1.42 | − | +2.36 |
| Chr. | GBAA_3313 | *hypF* | Hydrogenase maturation protein | +1.86 | − | +2.33 |
| Chr. | GBAA_3160 |  | Hypothetical protein | +1.20 | − | +2.3 |
| Chr. | GBAA_1087 |  | Hypothetical protein | +1.62 | − | +2.28 |
| Chr. | GBAA_1850-GBAA_1851 | *ilvB2-GBAA_1851* | Acetolactate synthase, large subunit-acetolactate synthase III, small subunit | − | − | +2.27 |
| Chr. | GBAA_4204 |  | Putative oxidoreductase | +0.77 | − | +2.26 |
| Chr. | GBAA_2627 | *cypA* | Cytochrome P450 | +0.89 | − | +2.25 |

| **TABLE S2** (Continued) | | | | **Log2(fold-change)^a^** | | |
| --- | --- | --- | --- | --- | --- | --- |
| **Genetic Element** | **Transcript locus tag** | **Gene names** | **Function** | **∆*xrrA*** | **∆*xrrB*** | **∆*xrrA***  **∆*xrrB*** |
| Chr. | GBAA_5607 |  | Hypothetical protein | +1.13 | − | +2.24 |
| Chr. | GBAA_2632 |  | Putative cytochrome P450 | +0.85 | − | +2.23 |
| Chr. | GBAA_0707-GBAA_0708 |  | Hypothetical protein-sodium/solute symporter | +1.14 | − | +2.22 |
| Chr. | GBAA_3159 |  | Hypothetical protein | +1.28 | − | +2.22 |
| Chr. | GBAA_0658 |  | Putative oligopeptide ABC transporter | +2.62 | +1.25 | +2.22 |
| Chr. | GBAA_4761 |  | Putative enoyl-CoA hydratase/isomerase | +0.85 | − | +2.19 |
| Chr. | GBAA_4760 | *etfB* | Electron transfer flavoprotein | +1.11 | − | +2.18 |
| Chr. | GBAA_3734 |  | Hypothetical protein | − | − | +2.17 |
| Chr. | GBAA_0657 |  | Putative oligopeptide ABC transporter | +2.64 | +1.42 | +2.13 |
| Chr. | GBAA_1751 | *asnO2* | Asparagine synthetase | − | − | +2.11 |
| Chr. | GBAA_4996 |  | Putative ABC transporter | − | − | +2.09 |
| Chr. | GBAA_2183 |  | Putative neutral metalloprotease | +1.22 | − | +2.08 |
| Chr. | GBAA_5532 | *nuoN* | NADH dehydrogenase (ubiquinone) | +1.45 | − | +2.06 |
| Chr. | GBAA_4915 | *acsA* | Acetyl-CoA synthetase | +0.95 | − | +2.04 |
| Chr. | GBAA_5498 |  | Putative DNA-binding protein | − | − | +2.02 |
| Chr. | GBAA_4707 |  | Hypothetical protein | − | − | +2 |
| Chr. | GBAA_1290 | *calY* | Camelysin | +2.72 | − | +1.97 |
| Chr. | GBAA_0385 |  | Putative chitinase B | +1.35 | +1.40 | +2.00 |
| Chr. | GBAA_4385 | *bfmbC* | 3-methyl-2-oxobutanoate dehydrogenase complex | -2.68 | -1.22 | -2.02 |
| Chr. | GBAA_0101 |  | YbxB protein | -0.97 | − | -2.02 |
| Chr. | GBAA_5301 |  | Sodium/alanine symporter | -1.28 | − | -2.05 |
| Chr. | GBAA_5240 | *ldh3* | L-lactate dehydrogenase | -1.67 | − | -2.08 |
| Chr. | GBAA_0614 |  | Proton/peptide symporter | -2.64 | − | -2.10 |
| Chr. | GBAA_5274 |  | Hypothetical protein | − | − | -2.12 |
| Chr. | GBAA_4969 | *glcU* | Glucose uptake protein | − | − | -2.13 |
| Chr. | GBAA_4069 |  | Hypothetical protein | − | − | -2.16 |
| Chr. | GBAA_0098 | *rplA* | Ribosomal protein L1 | -1.38 | − | -2.18 |
| Chr. | GBAA_5239 |  | Putative PTS sugar transporter subunit IIC | -2.01 | − | -2.18 |
| Chr. | GBAA_0034 | *abrB* | Transition state transcriptional regulator | -1.39 | − | -2.22 |

| **TABLE S2** (Continued) | | | | **Log2(fold-change)^a^** | | |
| --- | --- | --- | --- | --- | --- | --- |
| **Genetic Element** | **Transcript locus tag** | **Gene names** | **Function** | **∆*xrrA*** | **∆*xrrB*** | **∆*xrrA***  **∆*xrrB*** |
| Chr. | GBAA_2309 |  | Putative poly(A) polymerase PAP2 | -1.72 | − | -2.22 |
| Chr. | GBAA_4907 |  | Hypothetical protein | − | − | -2.23 |
| Chr. | GBAA_3451 |  | Putative serine/threonine transporter | -2.42 | − | -2.31 |
| Chr. | GBAA_0594 |  | ArsR Transcriptional Regulator | -2.50 | -2.65 | -2.44 |
| Chr. | GBAA_0510 | *pflA* | Pyruvate formate-lyase-activating enzyme | -2.29 | − | -2.46 |
| Chr. | GBAA_4384 | *bfmbAa* | 3-methyl-2-oxobutanoate dehydrogenase, alpha | -2.37 | -1.23 | -1.96 |
| Chr. | GBAA_0158 |  | Hypothetical protein | -2.35 | +0.96 | -1.98 |
| Chr. | GBAA_4382 | *bfmbB* | Dihydrolipoamide acetyltransferase | -2.56 | -1.12 | -1.13 |
| Chr. | GBAA_4383 | *bfmbAb* | 3-methyl-2-oxobutanoate dehydrogenase, beta subunit | -2.68 | -1.20 | -1.50 |
| Chr. | GBAA_2366-GBAA_2367 |  | Hypothetical protein-putative oxalate:formate antiporter | -4.23 | − | -1.48 |
| Chr. | GBAA_5690 |  | Putative holin | -4.63 | − | -1.20 |
| Chr. | GBAA_5689 |  | Putative membrane protein | -5.60 | − | -1.60 |
| pXO1 | GBAA_pXO1_0022 |  | Hypothetical protein | +2.40 | − | +3.29 |
| pXO1 | GBAA_pXO1_0153 |  | Hypothetical protein | -1.30 | − | -2.06 |
| pXO1 | GBAA_pXO1_0172 | *lef* | Anthrax lethal factor endopeptidase | − | − | -2.24 |
| pXO1 | GBAA_pXO1_0171 |  | Hypothetical protein | -0.96 | − | -2.47 |
| ^a^Hyphens indicate a nonsignificant difference in transcript expression. | | | | | | |
